# Future horizons in diabetes treatment: hypoglycemic activity of [1,2,4]triazino[2,3-*c*]quinazoline derivatives

**DOI:** 10.3389/fendo.2025.1638013

**Published:** 2025-09-18

**Authors:** Serhii Trzhetsynskyi, Inna Nosulenko, Anna Kinichenko, Dmytro Skoryna, Halyna Berest, Volodymyr Shvets, Oleksii Voskoboinik, Serhii Kovalenko, Pavlo Petakh, Oleksandr Kamyshnyi

**Affiliations:** ^1^ Department of Pharmacognosy, Pharmacology and Botany, Zaporizhzhia State Medical and Pharmaceutical University, Zaporizhzhia, Ukraine; ^2^ Department of Pharmaceutical, Organic and Bioorganic Chemistry, Zaporizhzhia State Medical and Pharmaceutical University, Zaporizhzhia, Ukraine; ^3^ Department of Clinical Pharmacy, Pharmacotherapy, Pharmacognosy and Pharmaceutical Chemistry, Zaporizhzhia State Medical and Pharmaceutical University, Zaporizhzhia, Ukraine; ^4^ Department of Biological Chemistry, Zaporizhzhia State Medical and Pharmaceutical University, Zaporizhzhia, Ukraine; ^5^ Department of Composite Materials, Chemistry and Technologies, National University “Zaporizhzhia Polytechnic”, Zaporizhzhia, Ukraine; ^6^ Research Institute of Chemistry and Geology, Oles Honchar Dnipro National University, Dnipro, Ukraine; ^7^ Department of Biochemistry and Pharmacology, Uzhhorod National University, Uzhhorod, Ukraine; ^8^ Department of Microbiology, Virology, and Immunology, I. Horbachevsky Ternopil National Medical University, Ternopil, Ukraine

**Keywords:** [1,2,4]triazino[2,3-c]quinazolines, hypoglycemic activity, diabetes mellitus, insulin resistance, molecular docking

## Abstract

Type 2 diabetes mellitus (T2DM) remains a significant and multifaceted challenge for modern healthcare. This issue becomes even more pressing during times of armed conflict and the subsequent recovery period, as research indicates an increased incidence of T2DM among combat veterans, largely due to post-traumatic stress disorder. Although numerous antidiabetic drugs are currently available, achieving optimal control of hyperglycemia continues to be problematic. In this context, and as part of a focused search for biologically active substances within the class of substituted and condensed [1,2,4]triazino[2,3-*c*]quinazolines, we explored the hypoglycemic effects of a newly synthesized series of such compounds. The study involved 21 synthesized compounds bearing the [1,2,4]triazino[2,3-*c*]quinazoline core. Experiments were conducted using white Wistar rats weighing between 260 and 280 grams. Prescreening of hypoglycemic activity was evaluated based on changes in blood glucose levels before and after compound administration by rats with normoglycemia. Compounds that demonstrated the most pronounced activity were selected for extended pharmacological evaluation using oral glucose tolerance test, adrenaline test, and rapid insulin tests in rats with dexamethasone-induced insulin resistance. Initial pharmacological screening under normoglycemic conditions showed that seven studied compounds significantly lowered blood glucose levels. Follow-up investigations validated the high hypoglycemic effect of 1,2,2-trimethyl-3-(3-methyl-2-oxo-2*H*- [1,2,4]triazino[2,3-*c*]quinazolin-6-yl)cyclopentane-1-carboxylic acid. Among the tested substances, compound 3-phenyl-6-(phenylamino)-2*H*-[1,2,4]triazino[2,3-*c*]quinazolin-2-one was the only one to exhibit moderate activity in the adrenaline tolerance test. None of the compounds enhanced insulin sensitivity in the liver or peripheral tissues. The findings suggest that substituted [1,2,4]triazino[2,3-*c*]quinazolines constitute a promising scaffold for the development of new hypoglycemic agents. 11β-Hydroxysteroid dehydrogenase is the most likely molecular target for lead-compound 1,2,2-trimethyl-3-(3-methyl-2-oxo-2*H*-[1,2,4]triazino[2,3-*c*]quinazolin-6-yl)cyclopentane-1-carboxylic acid.

## 1 Introduction

The issue of type 2 diabetes mellitus (T2DM) represents one of the most complex challenges for modern medical science (1–4). The issue of diabetes becomes particularly urgent during wartime and in the postwar period (5–8), as it has been shown that combat veterans are at increased risk of developing type 2 diabetes due to the consequences of post-traumatic stress disorder (9–12). Despite the continuous improvement of prevention strategies and pharmacological interventions, the incidence of this pathology demonstrates a persistent upward trend (13–16). Diabetes mellitus, as both a social and economic problem, is primarily associated with substantial societal expenditures—not only for the management of hyperglycemic conditions (17–19), but also for the treatment of a wide range of comorbidities (20–24). Furthermore, the high rate of disability associated with diabetes significantly increases the financial burden related to the support of individuals who have lost their ability to work (25–27). The current therapeutic paradigm for T2DM involves the early use of hypoglycemic agents in order to prevent disease progression (13, 28–30). The arsenal of drugs used to manage hyperglycemic states in patients with T2DM is fairly extensive and includes sulfonylurea derivatives, thiazolidinediones, and biguanides, among others (13, 31). Based on their mechanisms of action, additional agents include α-glucosidase inhibitors, dipeptidyl peptidase-4 (DPP-4) inhibitors, glucagon-like peptide-1 (GLP-1) receptor agonists, and prandial glucose regulators (13, 32–34).

Despite the availability of numerous agents in this therapeutic group, the problem of effectively treating hyperglycemia remains unresolved (35–38). This is due to the diversity of pathological manifestations of hyperglycemia, combined with the individual physiological characteristics of patients, which necessitates the development of drugs with improved pharmacotherapeutic profiles (39–41). The search for novel hypoglycemic agents is being actively pursued among various classes of organic compounds (42), including quinazoline and triazine derivatives (43–46).

Particular attention in modern approaches to the treatment of T2DM is directed toward metformin—a biguanide that has long remained the “gold standard” of first-line pharmacotherapy (47–50). Its high efficacy in reducing blood glucose levels is complemented by a favorable safety profile, a low risk of hypoglycemia, and beneficial effects on body weight (51–54). The primary mechanism of action of metformin involves the suppression of hepatic glucose production, enhancement of peripheral insulin sensitivity, and improvement of glucose uptake (55–57). In addition to its antihyperglycemic properties, metformin exhibits several pleiotropic effects, including anti-inflammatory, cardioprotective, and potentially neuroprotective actions (58–61). Recent studies also suggest its potential role in reducing the risk of certain malignancies (62–65). In the context of wartime and postwar periods, when a significant proportion of patients present with comorbid psycho-emotional and somatic disorders, metformin may play a pivotal role in comprehensive therapy (66–69). Its capacity to modulate metabolic and inflammatory pathways positions metformin as a promising agent not only in the management of hyperglycemia but also in addressing systemic consequences of stress and chronic inflammation (70–73).

In this context, special interest is being directed toward novel heterocyclic compounds, particularly [1,2,4]triazino[2,3-c]quinazoline derivatives (74–77). These molecules exhibit promising hypoglycemic and anti-inflammatory properties, making them attractive candidates for further investigation as multi-target agents in the treatment of T2DM, especially in patients with stress-related metabolic disturbances (78–81).

*Aim.* In view of the above, and within the framework of a targeted search for biologically active agents among substituted [1,2,4]triazino[2,3-*c*]quinazolines, we investigated the hypoglycemic activity of a series of compounds that are derivatives of abovementioned heterocyclic core.

## 2 Materials and methods

### 2.1 Studied compounds

**Figure 1:**
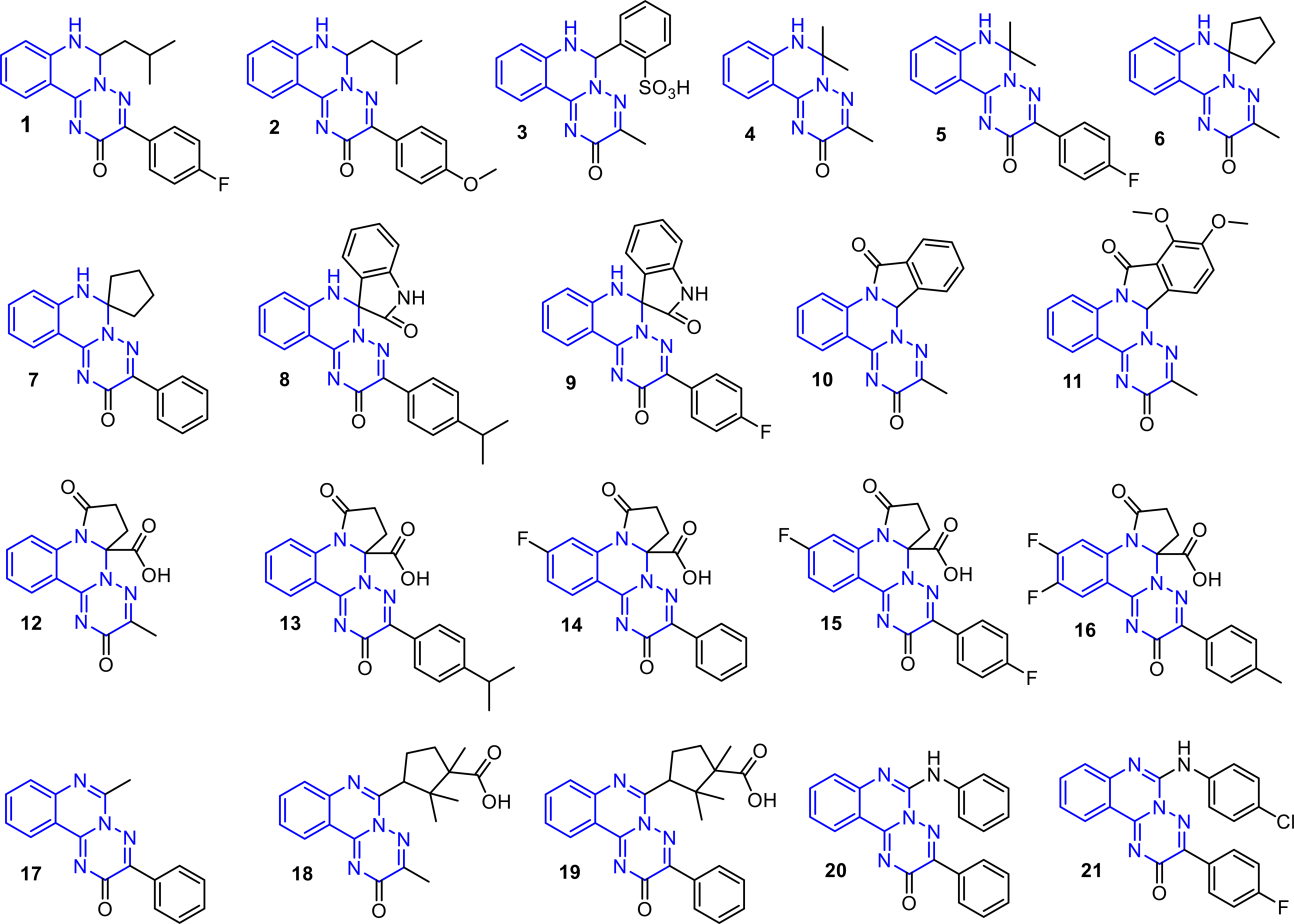
Structures of studied compounds.

A total of 21 compounds (**Figure 1**) containing the [1,2,4]triazino[2,3-*c*]quinazoline fragment were selected for the investigation of hypoglycemic activity. These compounds were obtained using previously described methods (77, 82–85) and exhibited identical spectral and physical characteristics. Selected compounds differ in the substituents at positions 3 and 6, the degree of saturation of the pyrimidine fragment, and the presence or absence of a fused or spiro-fused moiety. Such structural diversity provides a rational basis for identifying promising classes of compounds for further investigation.

### 2.2 Pharmacological studies

247 White male Wistar rats, each weighing between 260 and 280 g and aged 3.5 months, were employed in the experimental investigations. These animals were procured from the “Biomodelservis” nursery and maintained on a standard diet under a regular light–dark cycle, with unrestricted access to food and water. All experimental procedures were conducted in strict accordance with the “Regulations on the Use of Animals in Biomedical Research” (86). After a quarantine period, the individually identified animals were randomly allocated into groups of six male rats, ensuring uniformity in body weight (within a ±15% range) and the absence of external disease indicators.

#### 2.2.1 Preliminary screening

Prior to the commencement of the experiments, the rats were fasted overnight, and each animal was weighed. The test substances were administered intragastrically in either an aqueous solution or as a finely dispersed suspension stabilized with Tween 80, at a dosage of 50 mg/kg. The hypoglycemic potential of the synthesized compounds was determined by assessing the alterations in blood glucose levels before and following administration. For each compound, glucose levels were measured in six rats at 2-, 4-, 6-, and 8-hours post-administration. The evaluation of the potential hypoglycemic activity was based on the observed change in blood glucose concentration after a single oral dose, with measurements obtained via a “One Touch Select” blood glucose meter. A dynamic area under the curve (AUC) was calculated, where the time intervals (0, 2, 4, 6, and 8 hours) served as the z-coordinate and the percentage decrease in glucose levels as the y-coordinate.

#### 2.2.2 Induction of primary insulin resistance

Primary insulin resistance was induced by administering daily intramuscular injections of dexamethasone at a dosage of 0.125 mg/kg over a period of 13 days (87, 88). The studied compounds in dose 10 mg/kg were administrated daily simultaneously with injection of dexamethasone. Control groups of animals were also administered dexamethasone at the same dose and period, but instead of the suspension of the studied substance, they were administered an equivalent volume of water. The glucose homeostasis was then assessed by evaluating basal glycemia and carbohydrate tolerance. This assessment was performed using an oral glucose tolerance test, as well as adrenaline tests and rapid insulin test (87, 88). As reference standards, “Metformin” (administered at doses of 50 mg/kg) and “Gliclazide” (administered at 50 mg/kg) were used.

#### 2.2.3 Oral glucose tolerance test

Glucose was administered intragastrically at a dosage of 3 g/kg using a noninvasive probe. Blood samples for glucose determination were collected immediately before administration and subsequently at 15, 30, 60, and 120 minutes thereafter. A dynamic area under the curve (AUC) was calculated, where the time intervals (0, 0.25, 0.5, 1, 1.5 hours) served as the z-coordinate and the percentage increase in glucose levels as the y-coordinate.

#### 2.2.4 Adrenaline test

Rats received an intragastric dose of a 0.18% adrenaline solution at 0.5 mg/kg. Blood samples were collected for glucose analysis immediately before the administration, and at 30 and 90 minutes after the dose.

#### 2.2.5 Rapid Insulin test

Insulin was administered intraperitoneally at a dosage of 1 unit/kg. Glucose levels were measured immediately before and 30 minutes after injection.

#### 2.2.6 Statistical analysis

Data were processed using standard statistical software packages, specifically “Microsoft Office Excel 2003” and “STATISTICA^®^ for Windows 6.0” (StatSoft Inc., № AXXR712D833214FAN5). For each parameter, the arithmetic mean (M) and the standard error of the mean (± m) were calculated. The Mann-Whitney test was performed to prove the difference between studied groups of animals. The null hypothesis was rejected when the statistical criterion yielded a value of p < 0.05.

### 2.3 Docking study

Docking was carried out using the CB-Dock service (89, 90), which applies a protein-surface-curvature-based cavity detection approach to guide molecular docking with AutoDock Vina. α-Amylase (PDB ID: 1HNY), glucokinase (PDB ID: 1V4S), 11β-hydroxysteroid dehydrogenase (PDB ID: 2BEL), α-glucosidase (PDB ID: 3WY1), maltase-glucoamylase (PDB ID: 3TOP), fructose-1,6-bisphosphatase (PDB ID: 2JJK), and PPAR-γ (PDB ID: 2PRG) models were used as possible molecular targets.

## 3 Results

### 3.1 Pharmacological studies

At the initial stage, in order to identify promising candidates for further in-depth investigation of hypoglycemic activity, the blood glucose-lowering effect of a series of synthesized compounds was assessed using a normoglycemic model. The selection of doses for the experimental compounds was based on the efficacy of structurally related drugs or those with similar mechanisms of hypoglycemic action, as recommended by established guidelines. Based on these considerations, a dose of 50 mg/kg was chosen. Each compound was tested on a group of six rats during the screening phase. To evaluate the glucose-lowering effect over time, the percentage reduction in blood glucose was measured every two hours following oral administration of the test compound. Based on these data, the area under the curve (AUC) was calculated for the mean percentage decrease in blood glucose over time. The results of the hypoglycemic activity assessment in the normoglycemic model (**Table 1**) indicated that compounds 3, 12, and 17–21 demonstrated ability to reduce blood glucose levels in conditions of normoglycemic test.

**Table 1:** Effect of the tested compounds on blood glucose levels in rats under normoglycemic conditions.

Compound	AUC, %↓*h.	Compound	AUC, %↓*h.	Compound	AUC, %↓*h.
1	83.62	8	12.51	15	155.1
2	105.3	9	54.92	16	33.71
3	271.3	10	25.30	17	266.6
4	17.36	11	185.1	18	395.0
5	125.7	12	284.1	19	220.5
6	106.0	13	126.1	20	265.1
7	34.62	14	126.1	21	238.4

Based on the results of the primary screening of hypoglycemic activity, compounds 3, 17, 18, and 20 were selected for further in-depth investigation using a dexamethasone-induced diabetes model. The selection of compounds was based on their pronounced glucose-lowering activity under normoglycemic conditions, as well as their belonging to different classes of triazino[2,3-c]quinazoline derivatives. Specifically, compound 17 contains the simplest substituent at 6th position, compound 3 incorporates pharmacophoric sulfo-group, compound 18 bears a camphoric acid moiety, and compound 20 can be considered a structural analogue of biguanides. This model reproduces key pathological features such as impaired secretory function of pancreatic β-cells, development of insulin resistance, reduced carbohydrate tolerance, and decreased sensitivity of peripheral tissues to insulin action. Glucose homeostasis parameters were assessed using the oral glucose tolerance test (OGTT), the adrenaline test, and the short insulin test. These tests allow for the evaluation of basal glycemia, insulinemia, and carbohydrate tolerance. The results of the studies are presented in0 **Tables 2**–**4**.

**Table 2:** Hypoglycemic activity of the synthesized compounds in the glucose tolerance model.

Compound	Glucose level, mmol/l					AUC, ↑%*h
	Initial	15 min	30 min	60 min	90 min	
Control	4,6 ± 0,1	10,8 ± 0,1	11,0 ± 0,2	10,2 ± 0,2	7,3 ± 0,2	162.6
Metformin^**^	5,3 ± 0,3	12,1 ± 0,3^*^	11,3 ± 0,9	9,1 ± 0,4^*^	7,2 ± 0,6	118.7
Gliclazide^**^	4,0 ± 0,3	7,5 ± 0,8^*^	9,8 ± 1,2	10,4 ± 1,5	6,1 ± 0,3^*^	167.3
3^***^	5,5 ± 0,3^*^	10,0 ± 0,3^*^	9,1 ± 0,9^*^	7,9 ± 0,8	5,9 ± 0,4^*^	69.83
17^***^	5,1 ± 0,2^*^	8,5 ± 0,4^*^	10,2 ± 0,7	8,7 ± 1,1	5,4 ± 0,2^*^	89.10
18^***^	5,1 ± 0,3	7,2 ± 0,2^*^	7,7 ± 0,2^*^	7,4 ± 0,4*	5,0 ± 0,1^*^	53.16
20^***^	4,9 ± 0,2	8,9 ± 0,2^*^	11,2 ± 0,5	8,7 ± 0,8	7,0 ± 0,5	117.0

^*^р≤0.05 in comparison to control group of rats; ^**^in dose 50 mg/kg; ^***^in dose 10 mg/kg.

**Table 3:** Hypoglycemic activity of the synthesized compounds in the adrenaline tolerance test model.

Compound	Glucose level, mmol/l			Increase in glucose level, %	
	Initial	30 min	90 min	30 min	90 min
Control	5,1 ± 0,3	12,0 ± 0,6	1,6 ± 0,4^a^	139,1 ± 9,3	333,2 ± 22,4
Metformin^**^	5,7 ± 0,2	6,8 ± 0,2^*^	10.8 ± 0.3^*^	21.5 ± 3.3	93.1 ± 4.7
Gliclazide^**^	6.3 ± 0.2^*^	7.9 ± 0.2^*^	10.5 ± 0.3^*^	29.1 ± 5.9	66.8 ± 4.7
17^***^	5,0 ± 0,1	11,6± 0,7	20,5± 0,4	131,3 ± 10,1	311,1 ± 18,2
18^***^	5,0 ± 0,1	13,4 ± 0,4	19,3 ± 0,8^*^	170,8 ± 7,0	288,8 ± 15,0
20^***^	5,1 ± 0,2	12,0± 0,6	17,0± 0,6^*^	135,7 ± 12,1	235,2 ± 19,0

^*^р≤0.05 in comparison to control group of rats; ^**^in dose 50 mg/kg; ^***^in dose 10 mg/kg.

**Table 4:** Hypoglycemic activity of the synthesized compounds in the rapid insulin test.

Compound	Glucose level, mmol/l		Decrease in glucose level, %.
	Initial	30 min	30 min
Control	4,9 ± 0,2	2,6 ± 0,1	46,6 ± 1,4
Metformin^**^	5,2 ± 0,2	3,5 ± 0,3	33,4 ± 4,6
Gliclazide^**^	5,5 ± 0,3	3,9 ± 0,2^*^	30,3 ± 1,4
3^***^	5,0 ± 0,1	2,3 ± 0,1	52,8 ± 3,2
17^***^	5,0 ± 0,1	2,6 ± 0,2^*^	48,6 ± 2,5
18^***^	5,0 ± 0,3	2,8 ± 0,1	43,5 ± 4,0
20***	4,8 ± 0,1	2,9 ± 0,1	38,1 ± 3,2

^*^р≤0.05 in comparison to control group of rats; ^**^in dose 50 mg/kg; ^***^in dose 10 mg/kg.

As can be seen, the obtained results confirmed the data from the primary pharmacological screening regarding the pronounced hypoglycemic activity of compound 18. In the group of animals treated with compound 18, the average increase in blood glucose levels at 15, 30, 60, and 90 minutes after glucose loading increased to 42.8%, 52.4%, 46.4%, and –1.7%, respectively. Moreover, the area under the glucose-time curve (AUC) was the lowest among all tested groups, reaching 53.16%*h (**Table 2**). Hypoglycemic activity was also observed for compound 3. As shown by the results (**Table 3**), compound 20 was the only one among the tested substances to exhibit moderate hypoglycemic activity under the conditions of this model.

The short insulin tolerance test was performed to evaluate the sensitivity of both the liver and peripheral tissues to insulin. Analysis of the obtained results demonstrated that none of the tested compounds were capable of enhancing liver or peripheral tissue sensitivity to insulin. The average reduction in blood glucose levels 30 minutes after insulin administration in all groups treated with the test compounds showed only minor differences compared to the control group (**Table 4**).

### 3.2 Docking studies

Considering that biological studies have identified a promising hypoglycemic agent—namely, 1,2,2-trimethyl-3-(3-methyl-2-oxo-2*H*-[1,2,4]triazino[2,3-*c*]quinazolin-6-yl)cyclopentane-1-carboxylic acid (compound 18)—a investigation was initiated to determine the compound’s potential molecular mechanism of action. To achieve this goal, preliminary molecular docking studies were performed for compound 18 against the most common molecular targets of hypoglycemic drugs: α-amylase (PDB ID: 1HNY), glucokinase (PDB ID: 1V4S), 11β-hydroxysteroid dehydrogenase (PDB ID: 2BEL), α-glucosidase (PDB ID: 3WY1), maltase-glucoamylase (PDB ID: 3TOP), fructose-1,6-bisphosphatase (PDB ID: 2JJK), and PPAR-γ (PDB ID: 2PRG).

Docking was carried out using the CB-Dock service (89, 90), which applies a protein-surface-curvature-based cavity detection approach to guide molecular docking with AutoDock Vina. For each target, five distinct binding cavities were identified, and AutoDock Vina affinity scores were calculated for each site. **Table 5** summarizes the characteristics of the ligand–target complexes with the highest predicted binding affinities.

**Table 5:** The results of the docking study.

Molecular target	Cavity volume	Center (x, y, z)	Docking size (x, y, z)	Autodock vina affinity scores (kcal/mol)
1HNY	2521	-1, 41, 21	31, 21, 21	-8.4
1V4S	872	23, 5, 72	21, 21, 21	-8.3
2BEL	2449	-4, 22, -17	21, 28, 21	-10.6
3WY1	1358	15, -3, -4	27, 21, 21	-9.1
3TOP	2472	25, 14, -33	21, 28, 21	-9.0
2JJK	683	-56, 6, -65	21, 21, 21	-9.3
2PRG	6496	19, 26, 26	35, 34, 35	-9.3

The results indicate that compound 18 exhibits a notable predicted affinity for all the tested molecular targets. The highest affinity was observed for 11β-hydroxysteroid dehydrogenase (PDB ID: 2BEL), with an AutoDock Vina score of –10.6 kcal/mol. To better understand the interaction patterns between compound 18 and the molecular targets, docking results were visualized, allowing identification of the specific amino acid residues involved in ligand binding and the nature of these interactions (**Table 6**).

**Table 6:** The nature of amino acid moieties involved in formation of molecular target-ligand complex and nature of interactions.

Molecular target	Amino acid moiety (chain) and type of interaction
1HNY	ILE235(A)^9^, LEU162(A)^9^, HIS20(A)^10^, LYS200(A)^6^, ALA198(A)^6^, VAL234(A)^13^, GLU233(A)^13^, ASP300(A)^13^, TRP59(A)^13^, TRP58(A)^13^, TYR62(A)^13^, HIS299(A)^13^, ASP197(A)^13^, ARG195(A)^13^, HIS101(A)^13^, THR163(A)^13^, TYR151(A)^13^
1V4S	LYS414(A)^6^, SER455(A)^2^, LEU415(A)^13^, SER411(A)^13^, ASP409(A)^13^, GLY410(A)^13^, SER151(A)^13^, ASP78(A)^13^, ASP205(A)^13^, THR149(A)^13^, ILE225(A)^13^, GLY81(A)^13^, GLY227(A)^13^, THR82(A)^13^, THR228(A)^13^
2BEL	ILE46(B)^1^, GLY47(B)^1^, ASN119(B)^1^, ALA223(D)^9^, ILE121(B)^6^, GLY45(B)^2^, LEU217(B)^13^, GLY216(B)^13^, ILE218(B)^13^, THR220(B)^13^, HIS120(B)^13^, LYS44(B)^13^, GLY41(B)^13^, THR222(B)^13^, THR122(B)^13^, ASN123(B)^13^, THR124(B)^13^
2JJK	SER45(C)^1^, PRO188(D)^6^, ARG49(D)^6^, PRO188(C)^6^, ARG49(C)^6^, SER45(D)^13^, LEU186(D)^13^, SER46(D)^13^, ALA189(D)^13^, PRO188(A)^13^, PRO188(B)^13^, ALA189(A)^13^, ALA189(B)^13^, ALA189(C)^13^, LEU186(C)^13^, SER46(C)^13^
2PRG	LYS457(B)^6^, ILE456(B)^6^, LEU453(B)^6^, MET463(B)^6^, LEU465(B)^11^, SER464(B)^13^, GLN286(B)^13^, PHE282(B)^13^, VAL450(B)^13^, TYR473(B)^13^, ASP475(B)^13^, GLN454(B)^13^, GLN470(B)^13^, LYS474(B)^13^
3TOP	ASP1526(B)^7,8^, PHE1560(B)^10^, TRP1355(B)^10^, PHE1559(B)^10^, TYR1251(B)^10^, THR1586(B)^13^, MET1421(B)^13^, ARG1510(B)^13^, TRP1369(B)^13^, PHE1427(B)^13^, LYS1460(B)^13^, ASP1157(B)^13^, PRO1159(B)^13^, ILE1587(B)^13^
3WY1	ASN46(B)^1^, ASP346(A)^7,8^, ALA349(A)^6^, ARG450(B)^12^, GLN439(B)^2^, HIS348(A)^4^, GLN531(A)^13^, ASN443(A)^13^, SER44(B)^13^, ASP441(A)^13^, ALA444(A)^13^, ALA454(B)^13^, ASN447(B)^13^, ALA451(B)^13^, LYS352(A)^13^, PRO442(B)^13^, ASP440(B)^13^

^1^conventional hydrogen bond, ^2^carbon hydrogen bond, ^3^π-anion interaction, ^4^π-donor hydrogen bond, ^5^π-π stacked interaction, ^6^π-alkyl interaction, ^7^attractive charge, ^8^π-anion interaction, ^9^π-σ interaction, ^10^π-π T-shaped interaction, ^11^unfavorable donor-donor interaction, ^12^unfavorable positive-positive interaction ^13^van der Waals interaction,

As the data show, conventional hydrogen bonds were observed in only three complexes: 2BEL, 2JJK, and 3WY1. This type of interaction is known to contribute significantly to the strength and stability of ligand binding.

**Figure 2:**
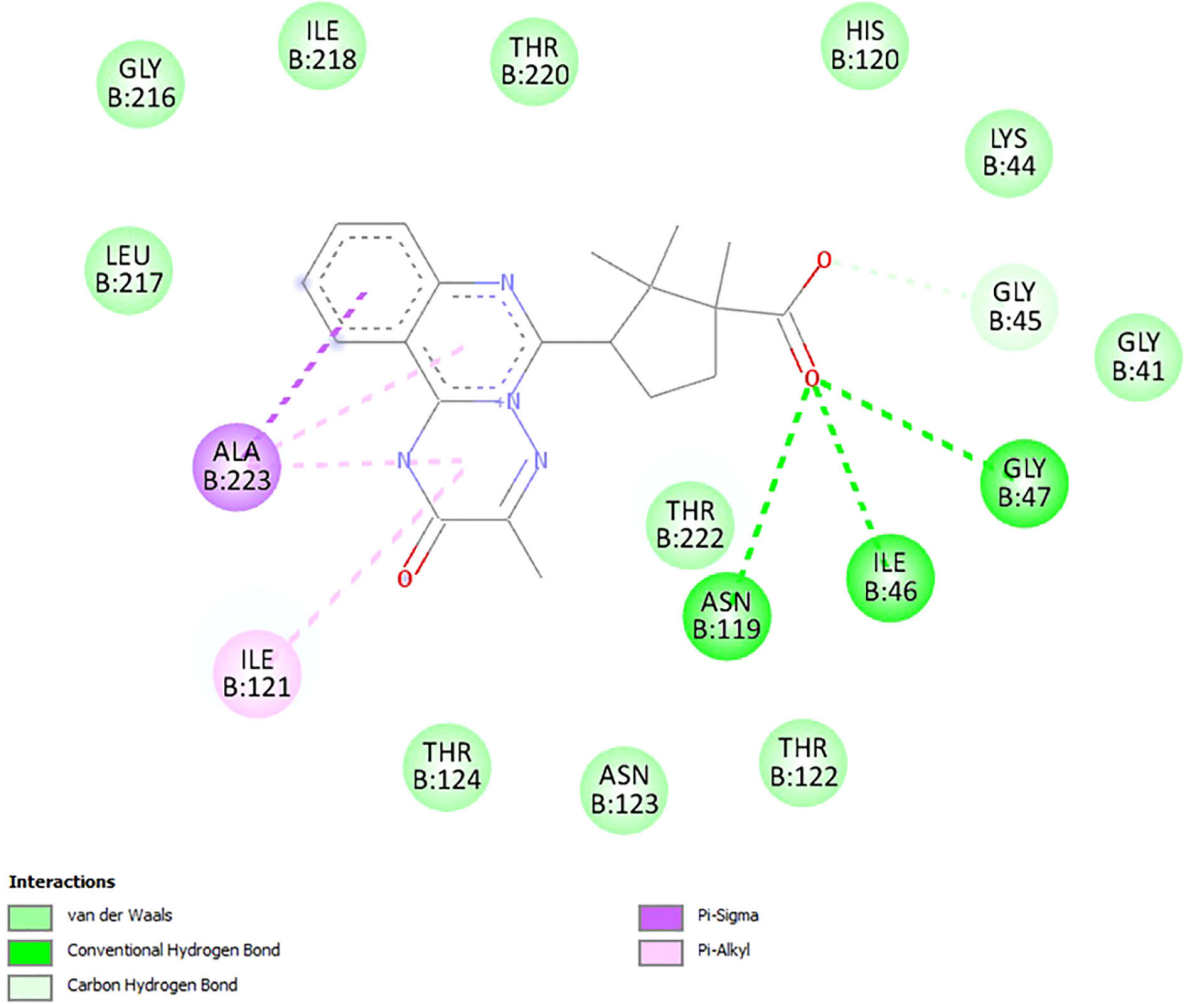
Visualization of docking study of compound 18 toward 11β-hydroxysteroid dehydrogenase (PDB ID: 2BEL).

Among these, the complex formed between compound 18 and 11β-hydroxysteroid dehydrogenase (PDB ID: 2BEL) stands out. It features three conventional hydrogen bonds involving the carboxyl group of the ligand and amino acid residues ILE46(B), GLY47(B), and ASN119(B), suggesting a particularly strong and specific interaction (**Figure 2**). In addition, the formation of a carbon–hydrogen bond with residue GLY45(B), a π–alkyl interaction with ILE121(B) and ALA223(B), and a π–σ interaction with ALA223(B) is also predicted, further contributing to the stability of the ligand–target complex. These findings are consistent with the highest calculated affinity of compound 18 toward 11β-hydroxysteroid dehydrogenase in comparison with other molecular targets. Thus, the obtained results suggest that 11β-hydroxysteroid dehydrogenase is the most likely molecular target for the lead compound 18. At the same time, it cannot be ruled out that the obtained compounds may exert hypoglycemic effects through interactions with multiple molecular targets. Considering the fact that multi-target drug development for the treatment of metabolic disorders is currently a trending area of research (91), this hypothesis underscores the relevance of experimentally elucidating the mechanism of the glucose-lowering action of the synthesized compounds.

## 4 Discussion

The findings of this study support the growing body of evidence that emphasizes the need for novel hypoglycemic agents with multi-target pharmacological properties (92–94). The promising glucose-lowering effects demonstrated by several [1,2,4]triazino[2,3-c]quinazoline derivatives, especially compound 18, underscore the therapeutic potential of this heterocyclic scaffold in the management of T2DM. The pronounced activity of these compounds in both normoglycemic and insulin-resistant models confirms their relevance for further pharmacological development. Notably, the docking results suggest that 11β-hydroxysteroid dehydrogenase may represent a key molecular target, which highlights the dual potential of these compounds in modulating both glucose metabolism and stress-related hormonal pathways.

Particular attention should be paid to metformin, a biguanide that remains the cornerstone of T2DM pharmacotherapy (95–97). Its efficacy, safety profile, and beneficial metabolic effects have made it the first-line treatment for decades (98–100). Beyond its antihyperglycemic action through hepatic glucose suppression and increased insulin sensitivity, metformin exerts pleiotropic effects—including anti-inflammatory, cardioprotective, and neuroprotective actions—which are especially valuable in patients with multiple comorbidities (101, 102). These properties highlight the need for new agents to retain or even expand upon these systemic effects, particularly in the postwar context, where stress-related metabolic disorders are prevalent (103–105).

An emerging area of interest in diabetes research is the role of gut microbiota (106, 107). Disruption of microbial balance—known as dysbiosis—has been implicated in the pathogenesis of insulin resistance and chronic inflammation (108–111). Recent studies suggest that the glucose-lowering effect of metformin is partly mediated through alterations in gut microbiota composition (112–114). Therefore, future investigation of [1,2,4]triazino[2,3-c]quinazoline derivatives should include an evaluation of their effects on the gut microbial ecosystem, particularly in stress-related and antibiotic-associated dysbiosis models.

Furthermore, the COVID-19 pandemic has highlighted the vulnerability of patients with T2DM to infectious diseases (115–117). The intersection between metabolic dysregulation and impaired immune responses creates a high-risk scenario for severe outcomes in viral infections such as SARS-CoV-2 (118–121). In this regard, the anti-inflammatory properties of several triazinoquinazoline derivatives may offer added value, potentially reducing cytokine-mediated complications during viral infections (122–125). Multifunctional agents that exert both metabolic and immunomodulatory actions could significantly improve outcomes in patients with dual metabolic and infectious burdens (126–128).

Genetic variability also plays a substantial role in the heterogeneity of T2DM presentation and treatment response (129–131). Single nucleotide polymorphisms (SNPs) in genes encoding insulin receptors, glucose transporters, inflammatory mediators, and drug-metabolizing enzymes can influence both disease progression and pharmacological outcomes (132–137). Understanding these genetic factors may enable the development of personalized therapeutic strategies that optimize efficacy and minimize adverse effects in individuals with T2DM (138–140), particularly those with comorbid conditions and increased susceptibility to infectious diseases (141–143). Integration of pharmacogenetic approaches may optimize the therapeutic application of newly developed agents, including the lead [1,2,4]triazino[2,3-c]quinazoline analogs, by aligning treatment choices with individual genetic profiles.

Importantly, T2DM rarely occurs in isolation and is often accompanied by multiple comorbidities such as obesity, cardiovascular disease, non-alcoholic fatty liver disease, depression, and post-traumatic stress disorder (144–146). These coexisting conditions complicate glycemic control and increase the risk of treatment failure (147–150). Therefore, the development of agents with broad systemic effects—including antioxidant, anti-inflammatory, and potentially psychotropic actions—is essential. The observed effects of some [1,2,4]triazino[2,3-c]quinazoline derivatives in this study suggest that these compounds may hold such potential and should be further examined in preclinical models of comorbidity.

Taken together, our results suggest that [1,2,4]triazino[2,3-c]quinazoline derivatives represent a promising chemical class for the development of novel hypoglycemic drugs. However, the complexity of T2DM requires an interdisciplinary approach that includes not only pharmacology, but also microbiology, genetics, immunology, and psychosomatic medicine (151–153). Further investigation is needed to elucidate the precise molecular targets and signaling pathways modulated by these derivatives (154–156). Given the involvement of chronic low-grade inflammation in T2DM and its comorbidities, special attention should be paid to the immunomodulatory properties of these compounds (157–160). Preliminary *in vitro* findings should be validated using *in vivo* models that accurately reflect the multifactorial nature of T2DM (161–163). Additionally, it is crucial to assess the safety profile, potential drug–drug interactions, and pharmacokinetic characteristics of these agents. Integration of omics technologies may help identify biomarkers predictive of response and toxicity (164–167). The inclusion of behavioral and cognitive endpoints in preclinical trials may also yield important insights into their psychotropic potential (168–170). Ultimately, translational research efforts will be essential to determine whether [1,2,4]triazino[2,3-c]quinazoline derivatives can address the unmet therapeutic needs of patients with T2DM and complex comorbid profiles.

## 5 Conclusions

Substituted and condensed [1,2,4]triazino[2,3-*c*]quinazolines have been demonstrated to represent a promising class of hypoglycemic agents. Preliminary screening under normoglycemic conditions revealed that 7 out of the 21 tested compounds exhibited a pronounced glucose-lowering effect. Selected compounds were further evaluated for their hypoglycemic activity using a model of primary insulin resistance. The oral glucose tolerance test indicated that 1,2,2-trimethyl-3-(3-methyl-2-oxo-2*H*-[1,2,4]triazino[2,3-*c*]quinazolin-6-yl)cyclopentane-1-carboxylic acid (compound 18) is a highly effective glucose-lowering agent. Conversely, in the adrenaline-induced hyperglycemia test, only 3-phenyl-6-(phenylamino)-2*H*-[1,2,4]triazino[2,3-*c*]quinazolin-2-one (compound 20) demonstrated a moderate hypoglycemic effect. None of the studied compounds enhanced insulin sensitivity in hepatic or peripheral tissues. Conducted docking study revealed that 11β-hydroxysteroid dehydrogenase is the most likely molecular target for lead-compound 1,2,2-trimethyl-3-(3-methyl-2-oxo-2*H*-[1,2,4]triazino[2,3-*c*]quinazolin-6-yl)cyclopentane-1-carboxylic acid (compound 18). These findings highlight the high potential of [1,2,4]triazino[2,3-*c*]quinazolin-2-one derivatives as a structural class for the development of novel hypoglycemic agents.

## Data Availability

The raw data supporting the conclusions of this article will be made available by the authors, without undue reservation.
