# Supplementary material for: Future horizons in diabetes treatment: hypoglycemic activity of [1,2,4]triazino[2,3-c]quinazoline derivatives
Source: Front Endocrinol (Lausanne). 2025 Sep 18;16:1638013. doi: 10.3389/fendo.2025.1638013 (PMC12488427; doi:10.3389/fendo.2025.1638013)
Supplement: Supplementary file 1 [file DataSheet1.pdf]

Supplementary material

Future Horizons in Diabetes Treatment: Hypoglycemic Activity Of [1,2,4]Triazino[2,3-c]Quinazoline Derivatives.

Serhii Trzhetsynskyi[1], Inna Nosulenko[1], Anna Kinichenko[1], Dmytro Skoryna[2], Halyna Berest[3], Volodymyr Shvets[4], Oleksii Voskoboinik[5], Serhii Kovalenko[6], Pavlo Petakh[7]*, Oleksandr Kamyshnyi[8]*

[1] Department of Pharmacognosy, Pharmacology and Botany, Zaporizhzhia State Medical and Pharmaceutical University, Mariia Pryimachenko Av, 26, Zaporizhzhia, Ukraine, 69035.

[2] Department of Pharmaceutical, Organic and Bioorganic Chemistry, Zaporizhzhia State Medical and Pharmaceutical University, Mariia Pryimachenko Av, 26, Zaporizhzhia, Ukraine, 69035.

[3] Department of Clinical Pharmacy, Pharmacotherapy, Pharmacognosy and Pharmaceutical Chemistry, Zaporizhzhia State Medical and Pharmaceutical University, Mariia Pryimachenko Av, 26, Zaporizhzhia, Ukraine, 69035.

[4] Department of Biological Chemistry, Zaporizhzhia State Medical and Pharmaceutical University, Mariia Pryimachenko Av, 26, Zaporizhzhia, Ukraine, 69035.

[5] Department of Composite Materials, Chemistry and Technologies, National University «Zaporizhzhia Polytechnic», Universytetska st. 64, Zaporizhzhia, Ukraine, 69011.

[6] Research Institute of Chemistry and Geology, Oles Honchar Dnipro National University, Nauky Ave. 72, Dnipro, Ukraine, 49010.

[7] Department of Biochemistry and Pharmacology, Uzhhorod National University, Uzhhorod, Ukraine

[8] Department of Microbiology, Virology, and Immunology, I. Horbachevsky Ternopil National Medical University, Ternopil, Ukraine, 46001

* Correspondence: pavlo.petakh@uzhnu.edu.ua , kamyshnyi_om@tdmu.edu.ua

S1 Effect of the tested compounds on blood glucose levels in rats under normoglycemic conditions (raw data).

S1.1 Compound 1.

Animal	Comp_1_0h	Comp_1_2h	Comp_1_4h	Comp_1_6h	Comp_1_8h
1	4,8	4,7	4	3,7	3
2	3,2	3,2	2,9	2,7	2,6
3	4,3	4,2	3,8	3,6	3,2
4	4,5	4,4	4,1	3,9	3,5
5	4,2	4,1	3,7	3,4	3,1
6	3,7	3,8	3,5	3,1	2,8

Descriptive statistics of Comp

	Comp_1_0h	Comp_1_2h	Comp_1_4h	Comp_1_6h	Comp_1_8h
Number of values	6	6	6	6	6
Minimum	3,200	3,200	2,900	2,700	2,600
Maximum	4,800	4,700	4,100	3,900	3,500
Range	1,600	1,500	1,200	1,200	0,9000
Mean	4,117	4,067	3,667	3,400	3,033
Std. Deviation	0,5776	0,5203	0,4320	0,4382	0,3141
Std. Error of Mean	0,2358	0,2124	0,1764	0,1789	0,1282

Test for normal distribution of Comp 1

	Comp_1_0h	Comp_1_2h	Comp_1_4h	Comp_1_6h	Comp_1_8h
Test for normal distribution					
Shapiro-Wilk test					
W	0,9549	0,9632	0,9083	0,9553	0,9926
P value	0,7794	0,8439	0,4255	0,7828	0,9946
Passed normality test (alpha=0.05)?	Yes	Yes	Yes	Yes	Yes
P value summary	ns	ns	ns	ns	ns
Kolmogorov-Smirnov test					
KS distance	0,2240	0,1922	0,1974	0,1760	0,1312
P value	>0,1000	>0,1000	>0,1000	>0,1000	>0,1000
Passed normality test (alpha=0.05)?	Yes	Yes	Yes	Yes	Yes
P value summary	ns	ns	ns	ns	ns
Number of values	6	6	6	6	6

S1.2 Compound 2.

Animal	Comp_2_0h	Comp_2_2h	Comp_2_4h	Comp_2_6h	Comp_2_8h
1	4,3	4,3	4,1	2,4	2,3
2	4,7	4,8	4,6	3,4	3
3	4,5	4,2	4,1	3,7	3,5
4	3,8	3,7	3,6	3,1	2,8
5	4,2	3,9	3,8	3	2,6
6	3,6	3,4	3,5	2,7	2,4

Descriptive statistics of Comp 2

	Comp_2_0h	Comp_2_2h	Comp_2_4h	Comp_2_6h	Comp_2_8h
Number of values	6	6	6	6	6
Minimum	3,600	3,400	3,500	2,400	2,300
Maximum	4,700	4,800	4,600	3,700	3,500
Range	1,100	1,400	1,100	1,300	1,200
Mean	4,183	4,050	3,950	3,050	2,767
Std. Deviation	0,4167	0,4930	0,4037	0,4680	0,4412
Std. Error of Mean	0,1701	0,2012	0,1648	0,1910	0,1801

Test for normal distribution of Comp 2

	Comp_2_0h	Comp_2_2h	Comp_2_4h	Comp_2_6h	Comp_2_8h
Test for normal distribution					
Shapiro-Wilk test					
W	0,9591	0,9870	0,9375	0,9900	0,9422
P value	0,8128	0,9805	0,6390	0,9892	0,6770
Passed normality test (alpha=0.05)?	Yes	Yes	Yes	Yes	Yes
P value summary	ns	ns	ns	ns	ns
Kolmogorov-Smirnov test					
KS distance	0,1826	0,1394	0,1885	0,1241	0,1472
P value	>0,1000	>0,1000	>0,1000	>0,1000	>0,1000
Passed normality test (alpha=0.05)?	Yes	Yes	Yes	Yes	Yes
P value summary	ns	ns	ns	ns	ns
Number of values	6	6	6	6	6

S1.3 Compound 3.

Animal	Comp_3_0h	Comp_3_2h	Comp_3_4h	Comp_3_6h	Comp_3_8h
1	3,4	1,8	1,9	2,3	2,7
2	3,6	1,9	2	2,4	2,9
3	4,5	2,4	2,2	2,2	3,1
4	3,2	1,8	2,1	2,5	2,6
5	4,2	2,6	2,3	2,7	3,3
6	3,7	2	2,1	2,3	2,6

Descriptive statistics of Comp 3

	Comp_3_0h	Comp_3_2h	Comp_3_4h	Comp_3_6h	Comp_3_8h
Number of values	6	6	6	6	6
Minimum	3,200	1,800	1,900	2,200	2,600
Maximum	4,500	2,600	2,300	2,700	3,300
Range	1,300	0,8000	0,4000	0,5000	0,7000
Mean	3,767	2,083	2,100	2,400	2,867
Std. Deviation	0,4926	0,3371	0,1414	0,1789	0,2875
Std. Error of Mean	0,2011	0,1376	0,05774	0,07303	0,1174

Test for normal distribution of Comp 3

	Comp_3_0h	Comp_3_2h	Comp_3_4h	Comp_3_6h	Comp_3_8h
Test for normal distribution					
Shapiro-Wilk test					
W	0,9423	0,8417	0,9818	0,9334	0,8946
P value	0,6777	0,1346	0,9600	0,6067	0,3430
Passed normality test (alpha=0.05)?	Yes	Yes	Yes	Yes	Yes
P value summary	ns	ns	ns	ns	ns
Kolmogorov-Smirnov test					
KS distance	0,2205	0,2643	0,1667	0,2119	0,2189
P value	>0,1000	>0,1000	>0,1000	>0,1000	>0,1000
Passed normality test (alpha=0.05)?	Yes	Yes	Yes	Yes	Yes
P value summary	ns	ns	ns	ns	ns
Number of values	6	6	6	6	6

S1.4 Compound 4.

Animal	Comp_4_0h	Comp_4_2h	Comp_4_4h	Comp_4_6h	Comp_4_8h
1	5,7	5,9	5,6	6,1	5,8
2	6,3	5,8	6,5	6,6	6,4
3	5,5	5,1	5,3	5,5	5,6
4	5,1	4,9	5,3	5,5	5,2
5	4,5	4,4	4,6	4,7	4,6
6	4,3	4,5	4,4	4,6	4,4

Descriptive statistics of Comp 4

	Comp_4_0h	Comp_4_2h	Comp_4_4h	Comp_4_6h	Comp_4_8h
Number of values	6	6	6	6	6
Minimum	4,300	4,400	4,400	4,600	4,400
Maximum	6,300	5,900	6,500	6,600	6,400
Range	2,000	1,500	2,100	2,000	2,000
Mean	5,233	5,100	5,283	5,500	5,333
Std. Deviation	0,7554	0,6356	0,7521	0,7772	0,7554
Std. Error of Mean	0,3084	0,2595	0,3070	0,3173	0,3084

Test for normal distribution of Comp 4

	Comp_4_0h	Comp_4_2h	Comp_4_4h	Comp_4_6h	Comp_4_8h
Test for normal distribution					
Shapiro-Wilk test					
W	0,9635	0,8983	0,9405	0,9334	0,9635
P value	0,8465	0,3638	0,6631	0,6065	0,8465
Passed normality test (alpha=0.05)?	Yes	Yes	Yes	Yes	Yes
P value summary	ns	ns	ns	ns	ns
Kolmogorov-Smirnov test					
KS distance	0,1675	0,1980	0,1755	0,1817	0,1675
P value	>0,1000	>0,1000	>0,1000	>0,1000	>0,1000
Passed normality test (alpha=0.05)?	Yes	Yes	Yes	Yes	Yes
P value summary	ns	ns	ns	ns	ns
Number of values	6	6	6	6	6

S1.5 Compound 5.

Animal	Comp_5_0h	Comp_5_2h	Comp_5_4h	Comp_5_6h	Comp_5_8h
1	5,4	5,3	5,1	4,2	4
2	4,6	4,2	4	3,3	2,9
3	5,7	4,9	4,1	3,9	3,7
4	4,2	4,1	3,7	3,2	2,7
5	4,6	4,3	3,9	3,4	2,9
6	3,9	3,7	3,5	3,1	2,6

Descriptive statistics of Comp 5

	Comp_5_0h	Comp_5_2h	Comp_5_4h	Comp_5_6h	Comp_5_8h
Number of values	6	6	6	6	6
Minimum	3,900	3,700	3,500	3,100	2,600
Maximum	5,700	5,300	5,100	4,200	4,000
Range	1,800	1,600	1,600	1,100	1,400
Mean	4,733	4,417	4,050	3,517	3,133
Std. Deviation	0,6919	0,5811	0,5577	0,4355	0,5750
Std. Error of Mean	0,2824	0,2372	0,2277	0,1778	0,2348

Test for normal distribution of Comp 5

	Comp_5_0h	Comp_5_2h	Comp_5_4h	Comp_5_6h	Comp_5_8h
Test for normal distribution					
Shapiro-Wilk test					
W	0,9329	0,9435	0,8503	0,8782	0,8434
P value	0,6024	0,6879	0,1582	0,2609	0,1391
Passed normality test (alpha=0.05)?	Yes	Yes	Yes	Yes	Yes
P value summary	ns	ns	ns	ns	ns
Kolmogorov-Smirnov test					
KS distance	0,2431	0,2462	0,2976	0,2723	0,3242
P value	>0,1000	>0,1000	>0,1000	>0,1000	0,0479
Passed normality test (alpha=0.05)?	Yes	Yes	Yes	Yes	No
P value summary	ns	ns	ns	ns	*
Number of values	6	6	6	6	6

S1.6 Compound 6

Animal	Comp_6_0h	Comp_6_2h	Comp_6_4h	Comp_6_6h	Comp_6_8h
1	5,8	4,9	4,4	4,2	4,3
2	5,2	5	4,6	4,3	4,5
3	4,4	4,2	3,8	3,5	3,7
4	4,6	4,4	3,9	3,6	3,8
5	5,1	4,8	4,5	4,2	4,4
6	4,2	3,9	3,3	3,2	3,6

Descriptive statistics of Comp 6

	Comp_6_0h	Comp_6_2h	Comp_6_4h	Comp_6_6h	Comp_6_8h
Number of values	6	6	6	6	6
Minimum	4,200	3,900	3,300	3,200	3,600
Maximum	5,800	5,000	4,600	4,300	4,500
Range	1,600	1,100	1,300	1,100	0,9000
Mean	4,883	4,533	4,083	3,833	4,050
Std. Deviation	0,5947	0,4367	0,5037	0,4590	0,3937
Std. Error of Mean	0,2428	0,1783	0,2056	0,1874	0,1607

Test for normal distribution of Comp 6

	Comp_6_0h	Comp_6_2h	Comp_6_4h	Comp_6_6h	Comp_6_8h
Test for normal distribution					
Shapiro-Wilk test					
W	0,9520	0,9250	0,9137	0,8690	0,8661
P value	0,7561	0,5422	0,4609	0,2221	0,2112
Passed normality test (alpha=0.05)?	Yes	Yes	Yes	Yes	Yes
P value summary	ns	ns	ns	ns	ns
Kolmogorov-Smirnov test					
KS distance	0,1831	0,2293	0,2352	0,2878	0,2373
P value	>0,1000	>0,1000	>0,1000	>0,1000	>0,1000
Passed normality test (alpha=0.05)?	Yes	Yes	Yes	Yes	Yes
P value summary	ns	ns	ns	ns	ns
Number of values	6	6	6	6	6

S1.7 Compound 7

Animal	Comp_7_0h	Comp_7_2h	Comp_7_4h	Comp_7_6h	Comp_7_8h
1	4,8	4,6	4,2	4,3	4,5
2	4,2	4,4	4,2	3,7	3,8
3	4,4	4,6	4,3	4,1	3,7
4	3,6	3,7	3,5	3,4	3,3
5	4,1	4	4,1	3,8	3,7
6	3,8	3,9	3,7	3,4	3,5

Descriptive statistics of Comp 7

	Comp_7_0h	Comp_7_2h	Comp_7_4h	Comp_7_6h	Comp_7_8h
Number of values	6	6	6	6	6
Minimum	3,600	3,700	3,500	3,400	3,300
Maximum	4,800	4,600	4,300	4,300	4,500
Range	1,200	0,9000	0,8000	0,9000	1,200
Mean	4,150	4,200	4,000	3,783	3,750
Std. Deviation	0,4278	0,3847	0,3225	0,3656	0,4087
Std. Error of Mean	0,1746	0,1571	0,1317	0,1493	0,1668

Test for normal distribution of Comp 7

	Comp_7_0h	Comp_7_2h	Comp_7_4h	Comp_7_6h	Comp_7_8h
Test for normal distribution					
Shapiro-Wilk test					
W	0,9839	0,8877	0,8467	0,9197	0,8771
P value	0,9692	0,3061	0,1481	0,5031	0,2559
Passed normality test (alpha=0.05)?	Yes	Yes	Yes	Yes	Yes
P value summary	ns	ns	ns	ns	ns
Kolmogorov-Smirnov test					
KS distance	0,1267	0,1984	0,2884	0,1861	0,2846
P value	>0,1000	>0,1000	>0,1000	>0,1000	>0,1000
Passed normality test (alpha=0.05)?	Yes	Yes	Yes	Yes	Yes
P value summary	ns	ns	ns	ns	ns
Number of values	6	6	6	6	6

S1.8 Compound 8

Animal	Comp_8_0h	Comp_8_2h	Comp_8_4h	Comp_8_6h	Comp_8_8h
1	6,5	6,1	6	6,1	6,5
2	5,5	5,7	5,9	6	5,8
3	5,4	5,3	5,5	5,7	5,4
4	5,3	5,2	5,4	5,6	5,6
5	4,9	5	5,1	5,3	5,2
6	5,6	5,5	5,4	5,5	5,7

Descriptive statistics of Comp 8

	Comp_8_0h	Comp_8_2h	Comp_8_4h	Comp_8_6h	Comp_8_8h
Number of values	6	6	6	6	6
Minimum	4,900	5,000	5,100	5,300	5,200
Maximum	6,500	6,100	6,000	6,100	6,500
Range	1,600	1,100	0,9000	0,8000	1,300
Mean	5,533	5,467	5,550	5,700	5,700
Std. Deviation	0,5317	0,3933	0,3391	0,3033	0,4472
Std. Error of Mean	0,2171	0,1606	0,1384	0,1238	0,1826

Test for normal distribution of Comp 8

	Comp_8_0h	Comp_8_2h	Comp_8_4h	Comp_8_6h	Comp_8_8h
Test for normal distribution					
Shapiro-Wilk test					
W	0,8904	0,9681	0,9213	0,9572	0,9158
P value	0,3202	0,8798	0,5151	0,7979	0,4756
Passed normality test (alpha=0.05)?	Yes	Yes	Yes	Yes	Yes
P value summary	ns	ns	ns	ns	ns
Kolmogorov-Smirnov test					
KS distance	0,2834	0,1641	0,2253	0,1720	0,2449
P value	>0,1000	>0,1000	>0,1000	>0,1000	>0,1000
Passed normality test (alpha=0.05)?	Yes	Yes	Yes	Yes	Yes
P value summary	ns	ns	ns	ns	ns
Number of values	6	6	6	6	6

S1.9 Compound 9

Animal	Comp_9_0h	Comp_9_2h	Comp_9_4h	Comp_9_6h	Comp_9_8h
1	3,9	4,1	3,7	3,6	2,9
2	4,3	4,7	4,4	3,9	3,2
3	4,1	4,1	4	3,7	3,4
4	4,5	4,6	4,3	4,1	3,3
5	3,8	4	3,5	3,3	2,9
6	4,2	4,4	4,1	3,7	3,4

Descriptive statistics of Comp 9

	Comp_9_0h	Comp_9_2h	Comp_9_4h	Comp_9_6h	Comp_9_8h
Number of values	6	6	6	6	6
Minimum	3,800	4,000	3,500	3,300	2,900
Maximum	4,500	4,700	4,400	4,100	3,400
Range	0,7000	0,7000	0,9000	0,8000	0,5000
Mean	4,133	4,317	4,000	3,717	3,183
Std. Deviation	0,2582	0,2927	0,3464	0,2714	0,2317
Std. Error of Mean	0,1054	0,1195	0,1414	0,1108	0,09458

Test for normal distribution of Comp 9

	Comp_9_0h	Comp_9_2h	Comp_9_4h	Comp_9_6h	Comp_9_8h
Test for normal distribution					
Shapiro-Wilk test					
W	0,9788	0,8885	0,9524	0,9728	0,8256
P value	0,9453	0,3105	0,7595	0,9106	0,0985
Passed normality test (alpha=0.05)?	Yes	Yes	Yes	Yes	Yes
P value summary	ns	ns	ns	ns	ns
Kolmogorov-Smirnov test					
KS distance	0,1503	0,2704	0,1667	0,1911	0,2227
P value	>0,1000	>0,1000	>0,1000	>0,1000	>0,1000
Passed normality test (alpha=0.05)?	Yes	Yes	Yes	Yes	Yes
P value summary	ns	ns	ns	ns	ns
Number of values	6	6	6	6	6

S1.10 Compound 10

Animal	Comp_10_0h	Comp_10_2h	Comp_10_4h	Comp_10_6h	Comp_10_8h
1	6,5	6,2	6,7	6,6	6,5
2	4,9	5,2	5,5	5,2	5,3
3	5,1	4,9	5,3	5,5	5,2
4	4,5	4,3	4,6	4,7	4,6
5	3,9	4	4,1	4,2	3,9
6	4,3	4,5	4,6	4,6	4,4

Descriptive statistics of Comp 10

	Comp_10_0h	Comp_10_2h	Comp_10_4h	Comp_10_6h	Comp_10_8h
Number of values	6	6	6	6	6
Minimum	3,900	4,000	4,100	4,200	3,900
Maximum	6,500	6,200	6,700	6,600	6,500
Range	2,600	2,200	2,600	2,400	2,600
Mean	4,867	4,850	5,133	5,133	4,983
Std. Deviation	0,9070	0,7868	0,9223	0,8524	0,9065
Std. Error of Mean	0,3703	0,3212	0,3765	0,3480	0,3701

Test for normal distribution of Comp 10

	Comp_10_0h	Comp_10_2h	Comp_10_4h	Comp_10_6h	Comp_10_8h
Test for normal distribution					
Shapiro-Wilk test					
W	0,9071	0,9365	0,9275	0,9317	0,9517
P value	0,4174	0,6311	0,5606	0,5934	0,7538
Passed normality test (alpha=0.05)?	Yes	Yes	Yes	Yes	Yes
P value summary	ns	ns	ns	ns	ns
Kolmogorov-Smirnov test					
KS distance	0,2318	0,1718	0,2185	0,1944	0,1967
P value	>0,1000	>0,1000	>0,1000	>0,1000	>0,1000
Passed normality test (alpha=0.05)?	Yes	Yes	Yes	Yes	Yes
P value summary	ns	ns	ns	ns	ns
Number of values	6	6	6	6	6

S1.11 Compound 11

Animal	Comp_11_0h	Comp_11_2h	Comp_11_4h	Comp_11_6h	Comp_11_8h
1	3,4	3,2	3	2,3	2,2
2	4,6	3,4	2,8	2,5	2,9
3	4,1	3,6	3,3	2,6	2,1
4	4,4	3,8	3,5	2,5	2,8
5	3,9	3,5	3,2	2,7	2,5
6	3,7	3,2	2,9	2,2	2,4

Descriptive statistics of Comp 11

	Comp_11_0h	Comp_11_2h	Comp_11_4h	Comp_11_6h	Comp_11_8h
Number of values	6	6	6	6	6
Minimum	3,400	3,200	2,800	2,200	2,100
Maximum	4,600	3,800	3,500	2,700	2,900
Range	1,200	0,6000	0,7000	0,5000	0,8000
Mean	4,017	3,450	3,117	2,467	2,483
Std. Deviation	0,4446	0,2345	0,2639	0,1862	0,3189
Std. Error of Mean	0,1815	0,09574	0,1078	0,07601	0,1302

Test for normal distribution of Comp 11

	Comp_11_0h	Comp_11_2h	Comp_11_4h	Comp_11_6h	Comp_11_8h
Test for normal distribution					
Shapiro-Wilk test					
W	0,9831	0,9343	0,9658	0,9496	0,9410
P value	0,9659	0,6139	0,8631	0,7368	0,6674
Passed normality test (alpha=0.05)?	Yes	Yes	Yes	Yes	Yes
P value summary	ns	ns	ns	ns	ns
Kolmogorov-Smirnov test					
KS distance	0,1390	0,1901	0,1708	0,2377	0,1730
P value	>0,1000	>0,1000	>0,1000	>0,1000	>0,1000
Passed normality test (alpha=0.05)?	Yes	Yes	Yes	Yes	Yes
P value summary	ns	ns	ns	ns	ns
Number of values	6	6	6	6	6

S1.12 Compound 12

Animal	Comp_12_0h	Comp_12_2h	Comp_12_4h	Comp_12_6h	Comp_12_8h
1	3,6	1,8	1,9	2	2,5
2	3,9	2,1	2	2,4	3,1
3	3,3	2	1,8	2,3	2,8
4	3,2	1,8	1,7	2	2,7
5	4,5	2,3	2,1	2,6	3,4
6	4,1	2	2,2	2,8	3,5

Descriptive statistics of Comp 12

	Comp_12_0h	Comp_12_2h	Comp_12_4h	Comp_12_6h	Comp_12_8h
Number of values	6	6	6	6	6
Minimum	3,200	1,800	1,700	2,000	2,500
Maximum	4,500	2,300	2,200	2,800	3,500
Range	1,300	0,5000	0,5000	0,8000	1,000
Mean	3,767	2,000	1,950	2,350	3,000
Std. Deviation	0,4967	0,1897	0,1871	0,3209	0,4000
Std. Error of Mean	0,2028	0,07746	0,07638	0,1310	0,1633

Test for normal distribution of Comp 12

	Comp_12_0h	Comp_12_2h	Comp_12_4h	Comp_12_6h	Comp_12_8h
Test for normal distribution					
Shapiro-Wilk test					
W	0,9579	0,9144	0,9819	0,9288	0,9373
P value	0,8032	0,4660	0,9606	0,5712	0,6377
Passed normality test (alpha=0.05)?	Yes	Yes	Yes	Yes	Yes
P value summary	ns	ns	ns	ns	ns
Kolmogorov-Smirnov test					
KS distance	0,1596	0,1874	0,1220	0,1956	0,1915
P value	>0,1000	>0,1000	>0,1000	>0,1000	>0,1000
Passed normality test (alpha=0.05)?	Yes	Yes	Yes	Yes	Yes
P value summary	ns	ns	ns	ns	ns
Number of values	6	6	6	6	6

S1.13 Compound 13

Animal	Comp_13_0h	Comp_13_2h	Comp_13_4h	Comp_13_6h	Comp_13_8h
1	3,4	2,5	2,2	2,3	2,7
2	4,6	1,9	2,2	2,4	3,1
3	3,6	1,9	2	2,3	2,8
4	4,1	2,1	2,3	2,6	3,3
5	3,5	2,2	2,4	2,5	2,9
6	4,2	2,8	2,9	3,1	3,4

Descriptive statistics of Comp 13

	Comp_13_0h	Comp_13_2h	Comp_13_4h	Comp_13_6h	Comp_13_8h
Number of values	6	6	6	6	6
Minimum	3,400	1,900	2,000	2,300	2,700
Maximum	4,600	2,800	2,900	3,100	3,400
Range	1,200	0,9000	0,9000	0,8000	0,7000
Mean	3,900	2,233	2,333	2,533	3,033
Std. Deviation	0,4733	0,3559	0,3077	0,3011	0,2805
Std. Error of Mean	0,1932	0,1453	0,1256	0,1229	0,1145

Test for normal distribution of Comp 13

	Comp_13_0h	Comp_13_2h	Comp_13_4h	Comp_13_6h	Comp_13_8h

Test for normal distribution

Shapiro-Wilk test					
W	0,9148	0,9023	0,8752	0,8139	0,9401
P value	0,4684	0,3874	0,2479	0,0780	0,6599
Passed normality test (alpha=0.05)?	Yes	Yes	Yes	Yes	Yes
P value summary	ns	ns	ns	ns	ns
Kolmogorov-Smirnov test					
KS distance	0,2369	0,2040	0,2476	0,2457	0,1827
P value	>0,1000	>0,1000	>0,1000	>0,1000	>0,1000
Passed normality test (alpha=0.05)?	Yes	Yes	Yes	Yes	Yes
P value summary	ns	ns	ns	ns	ns
Number of values	6	6	6	6	6

S1.14 Compound 14

Animal	Comp_14_0h	Comp_14_2h	Comp_14_4h	Comp_14_6h	Comp_14_8h
1	3,6	3,2	2,6	2,5	2,4
2	3,8	3,1	2,7	2,6	2,7
3	3,7	2,9	2,6	2,4	2,2
4	3,9	3,3	3,1	2,8	2,7
5	3,7	3,1	2,7	2,3	2,5
6	4,3	3,6	3,3	2,8	2,6

Descriptive statistics of Comp 14

	Comp_14_0h	Comp_14_2h	Comp_14_4h	Comp_14_6h	Comp_14_8h
Number of values	6	6	6	6	6
Minimum	3,600	2,900	2,600	2,300	2,200
Maximum	4,300	3,600	3,300	2,800	2,700
Range	0,7000	0,7000	0,7000	0,5000	0,5000
Mean	3,833	3,200	2,833	2,567	2,517
Std. Deviation	0,2503	0,2366	0,2944	0,2066	0,1941
Std. Error of Mean	0,1022	0,09661	0,1202	0,08433	0,07923

Test for normal distribution of Comp 14

	Comp_14_0h	Comp_14_2h	Comp_14_4h	Comp_14_6h	Comp_14_8h
Test for normal distribution					
Shapiro-Wilk test					
W	0,8466	0,9474	0,8045	0,9182	0,9124
P value	0,1478	0,7192	0,0645	0,4928	0,4522
Passed normality test (alpha=0.05)?	Yes	Yes	Yes	Yes	Yes
P value summary	ns	ns	ns	ns	ns
Kolmogorov-Smirnov test					
KS distance	0,2283	0,1696	0,3414	0,2040	0,1724
P value	>0,1000	>0,1000	0,0276	>0,1000	>0,1000
Passed normality test (alpha=0.05)?	Yes	Yes	No	Yes	Yes
P value summary	ns	ns	*	ns	ns
Number of values	6	6	6	6	6

S1.15 Compound 15

Comp_15_0h	Comp_15_2h	Comp_15_4h	Comp_15_6h	Comp_15_8h
Animal	3,9	3,8	2,9	2,8
1	3,2	2,6	2,6	2,4
2	3,2	3,1	3	2,8
3	4,2	3,6	3,1	3
4	3	2,6	2,2	2
5	3,5	3,3	2,6	2,3
6				

Descriptive statistics of Comp 15

	Comp_15_0h	Comp_15_2h	Comp_15_4h	Comp_15_6h	Comp_15_8h
Number of values	6	6	6	6	6
Minimum	3,600	3,000	2,600	2,200	2,000
Maximum	5,200	4,200	3,800	3,100	3,000
Range	1,600	1,200	1,200	0,9000	1,000
Mean	4,550	3,500	3,167	2,733	2,550
Std. Deviation	0,5541	0,4648	0,5007	0,3327	0,3782
Std. Error of Mean	0,2262	0,1897	0,2044	0,1358	0,1544

Test for normal distribution of Comp 15

	Comp_15_0h	Comp_15_2h	Comp_15_4h	Comp_15_6h	Comp_15_8h
Test for normal distribution					
Shapiro-Wilk test					
W	0,9475	0,9158	0,9134	0,9302	0,9373
P value	0,7202	0,4759	0,4589	0,5817	0,6373
Passed normality test (alpha=0.05)?	Yes	Yes	Yes	Yes	Yes
P value summary	ns	ns	ns	ns	ns
Kolmogorov-Smirnov test					
KS distance	0,2026	0,2407	0,2045	0,1918	0,2457
P value	>0,1000	>0,1000	>0,1000	>0,1000	>0,1000
Passed normality test (alpha=0.05)?	Yes	Yes	Yes	Yes	Yes
P value summary	ns	ns	ns	ns	ns
Number of values	6	6	6	6	6

S1.13 Compound 16

Animal	Comp_16_0h	Comp_16_2h	Comp_16_4h	Comp_16_6h	Comp_16_8h
1	3,1	3,2	3,3	2,5	2,4
2	3,8	3,9	3,9	3,2	2,8
3	3,6	3,8	4	2,6	2,5
4	4,1	4,2	4,6	3,5	3,3
5	3,8	4	4,1	3,1	2,6
6	4,3	4,4	4,5	3,7	3,4

Descriptive statistics of Comp 16

	Comp_16_0h	Comp_16_2h	Comp_16_4h	Comp_16_6h	Comp_16_8h
Number of values	6	6	6	6	6
Minimum	3,100	3,200	3,300	2,500	2,400
Maximum	4,300	4,400	4,600	3,700	3,400
Range	1,200	1,200	1,300	1,200	1,000
Mean	3,783	3,917	4,067	3,100	2,833
Std. Deviation	0,4167	0,4119	0,4676	0,4775	0,4227
Std. Error of Mean	0,1701	0,1682	0,1909	0,1949	0,1726

Test for normal distribution of Comp 16

	Comp_16_0h	Comp_16_2h	Comp_16_4h	Comp_16_6h	Comp_16_8h
Test for normal distribution					
Shapiro-Wilk test					
W	0,9577	0,9393	0,9388	0,9361	0,8771
P value	0,8018	0,6539	0,6494	0,6280	0,2561
Passed normality test (alpha=0.05)?	Yes	Yes	Yes	Yes	Yes
P value summary	ns	ns	ns	ns	ns
Kolmogorov-Smirnov test					
KS distance	0,1826	0,2218	0,1941	0,1858	0,2095
P value	>0,1000	>0,1000	>0,1000	>0,1000	>0,1000
Passed normality test (alpha=0.05)?	Yes	Yes	Yes	Yes	Yes
P value summary	ns	ns	ns	ns	ns
Number of values	6	6	6	6	6

S1.13 Compound 17

Animal	Comp_17_0h	Comp_17_2h	Comp_17_4h	Comp_17_6h	Comp_17_8h
1	5,7	3,3	2,1	2,3	2,7
2	4,5	3,8	2,4	2,9	3,2
3	4,3	3,4	2,2	2,8	3,5
4	4	3,2	2,3	2,4	2,8
5	3,8	3,1	1,9	2,2	2,9
6	4,7	3,4	2,5	2,3	3,1

Descriptive statistics of Comp 17

	Comp_17_0h	Comp_17_2h	Comp_17_4h	Comp_17_6h	Comp_17_8h
Number of values	6	6	6	6	6
Minimum	3,800	3,100	1,900	2,200	2,700
Maximum	5,700	3,800	2,500	2,900	3,500
Range	1,900	0,7000	0,6000	0,7000	0,8000
Mean	4,500	3,367	2,233	2,483	3,033
Std. Deviation	0,6723	0,2422	0,2160	0,2927	0,2944
Std. Error of Mean	0,2745	0,09888	0,08819	0,1195	0,1202

Test for normal distribution of Comp 17

	Comp_17_0h	Comp_17_2h	Comp_17_4h	Comp_17_6h	Comp_17_8h
Test for normal distribution					
Shapiro-Wilk test					
W	0,9122	0,9043	0,9826	0,8384	0,9580
P value	0,4508	0,4002	0,9637	0,1264	0,8043
Passed normality test (alpha=0.05)?	Yes	Yes	Yes	Yes	Yes
P value summary	ns	ns	ns	ns	ns
Kolmogorov-Smirnov test					
KS distance	0,2164	0,2786	0,1212	0,2787	0,1747
P value	>0,1000	>0,1000	>0,1000	>0,1000	>0,1000
Passed normality test (alpha=0.05)?	Yes	Yes	Yes	Yes	Yes
P value summary	ns	ns	ns	ns	ns
Number of values	6	6	6	6	6

S1.18 Compound 18

Animal	Comp_18_0h	Comp_18_2h	Comp_18_4h	Comp_18_6h	Comp_18_8h
1	3,8	2,3	1,9	2,2	2,5
2	3,9	2,3	2,1	2,3	2,8
3	3,5	2,1	1,9	1,7	2,9
4	4,4	2,8	2,3	2,4	3,1
5	4,1	2,4	2,1	2,5	3,3
6	3,6	2,2	1,9	2	2,8

Descriptive statistics of Comp 18

	Comp_18_0h	Comp_18_2h	Comp_18_4h	Comp_18_6h	Comp_18_8h
Number of values	6	6	6	6	6
Minimum	3,500	2,100	1,900	1,700	2,500
Maximum	4,400	2,800	2,300	2,500	3,300
Range	0,9000	0,7000	0,4000	0,8000	0,8000
Mean	3,883	2,350	2,033	2,183	2,900
Std. Deviation	0,3312	0,2429	0,1633	0,2927	0,2757
Std. Error of Mean	0,1352	0,09916	0,06667	0,1195	0,1125

Test for normal distribution of Comp 18

	Comp_18_0h	Comp_18_2h	Comp_18_4h	Comp_18_6h	Comp_18_8h
Test for normal distribution					
Shapiro-Wilk test					
W	0,9662	0,8687	0,8216	0,9428	0,9709
P value	0,8657	0,2210	0,0911	0,6820	0,8986
Passed normality test (alpha=0.05)?	Yes	Yes	Yes	Yes	Yes
P value summary	ns	ns	ns	ns	ns
Kolmogorov-Smirnov test					
KS distance	0,1466	0,2518	0,2929	0,1894	0,1917
P value	>0,1000	>0,1000	>0,1000	>0,1000	>0,1000
Passed normality test (alpha=0.05)?	Yes	Yes	Yes	Yes	Yes
P value summary	ns	ns	ns	ns	ns
Number of values	6	6	6	6	6

S1.19 Compound 19

Animal	Comp_19_0h	Comp_19_2h	Comp_19_4h	Comp_19_6h	Comp_19_8h
1	3	2,3	2,2	1,9	2,4
2	3,2	2,2	2,4	2,1	2,8
3	3,4	2,1	2,2	2	2,5
4	3,8	2,5	2,4	2,2	2,9
5	4,3	3,1	2,9	2,7	3,4
6	3,6	2,7	2,4	2,1	3,1

Descriptive statistics of Comp 19

	Comp_19_0h	Comp_19_2h	Comp_19_4h	Comp_19_6h	Comp_19_8h
Number of values	6	6	6	6	6
Minimum	3,000	2,100	2,200	1,900	2,400
Maximum	4,300	3,100	2,900	2,700	3,400
Range	1,300	1,000	0,7000	0,8000	1,000
Mean	3,550	2,483	2,417	2,167	2,850
Std. Deviation	0,4637	0,3710	0,2563	0,2805	0,3728
Std. Error of Mean	0,1893	0,1515	0,1046	0,1145	0,1522

Test for normal distribution of Comp 19

	Comp_19_0h	Comp_19_2h	Comp_19_4h	Comp_19_6h	Comp_19_8h
Test for normal distribution					
Shapiro-Wilk test					
W	0,9715	0,9321	0,7805	0,8275	0,9680
P value	0,9024	0,5961	0,0390	0,1024	0,8788
Passed normality test (alpha=0.05)?	Yes	Yes	No	Yes	Yes
P value summary	ns	ns	*	ns	ns
Kolmogorov-Smirnov test					
KS distance	0,1282	0,1894	0,3593	0,2860	0,1594
P value	>0,1000	>0,1000	0,0150	>0,1000	>0,1000
Passed normality test (alpha=0.05)?	Yes	Yes	No	Yes	Yes
P value summary	ns	ns	*	ns	ns
Number of values	6	6	6	6	6

S1.20 Compound 20

Animal	Comp_20_0h	Comp_20_2h	Comp_20_4h	Comp_20_6h	Comp_20_8h
1	4,3	3,2	2,2	2,6	2,9
2	4,8	3,1	2,2	2,5	3,2
3	4,5	3,9	2,5	2,4	2,6
4	4,2	3,5	2,5	2,2	2,5
5	4,7	3,8	2,1	2,6	3
6	4,1	3,1	1,9	2,7	3,2

Descriptive statistics of Comp 20

	Comp_20_0h	Comp_20_2h	Comp_20_4h	Comp_20_6h	Comp_20_8h
Number of values	6	6	6	6	6
Minimum	4,100	3,100	1,900	2,200	2,500
Maximum	4,800	3,900	2,500	2,700	3,200
Range	0,7000	0,8000	0,6000	0,5000	0,7000
Mean	4,433	3,433	2,233	2,500	2,900
Std. Deviation	0,2805	0,3559	0,2338	0,1789	0,2966
Std. Error of Mean	0,1145	0,1453	0,09545	0,07303	0,1211

Test for normal distribution of Comp 20

	Comp_20_0h	Comp_20_2h	Comp_20_4h	Comp_20_6h	Comp_20_8h
Test for normal distribution					
Shapiro-Wilk test					
W	0,9401	0,8585	0,9076	0,9334	0,8944
P value	0,6599	0,1839	0,4207	0,6067	0,3420
Passed normality test (alpha=0.05)?	Yes	Yes	Yes	Yes	Yes
P value summary	ns	ns	ns	ns	ns
Kolmogorov-Smirnov test					
KS distance	0,1827	0,2440	0,2234	0,2119	0,1774
P value	>0,1000	>0,1000	>0,1000	>0,1000	>0,1000
Passed normality test (alpha=0.05)?	Yes	Yes	Yes	Yes	Yes
P value summary	ns	ns	ns	ns	ns
Number of values	6	6	6	6	6

S1.21 Compound 21

Comp_21_0h	Comp_21_2h	Comp_21_4h	Comp_21_6h	Comp_21_8h
Animal	3,7	2,1	2,3	2,6
1	2,6	2,1	2,2	2,8
2	3,1	2,3	2,2	3
3	3,8	2,7	2,4	3,1
4	3	2,5	2	2,5
5	3,5	2,6	2,3	2,9
6				

Descriptive statistics of Comp 21

	Comp_21_0h	Comp_21_2h	Comp_21_4h	Comp_21_6h	Comp_21_8h
Number of values	6	6	6	6	6
Minimum	3,600	2,600	2,100	2,000	2,500
Maximum	4,700	3,800	2,700	2,400	3,100
Range	1,100	1,200	0,6000	0,4000	0,6000
Mean	4,050	3,283	2,383	2,233	2,817
Std. Deviation	0,4680	0,4622	0,2563	0,1366	0,2317
Std. Error of Mean	0,1910	0,1887	0,1046	0,05578	0,09458

Test for normal distribution of Comp 21

	Comp_21_0h	Comp_21_2h	Comp_21_4h	Comp_21_6h	Comp_21_8h
Test for normal distribution					
Shapiro-Wilk test					
W	0,8885	0,9423	0,9006	0,9266	0,9575
P value	0,3106	0,6782	0,3777	0,5544	0,8006
Passed normality test (alpha=0.05)?	Yes	Yes	Yes	Yes	Yes
P value summary	ns	ns	ns	ns	ns
Kolmogorov-Smirnov test					
KS distance	0,2034	0,1804	0,1989	0,2370	0,1585
P value	>0,1000	>0,1000	>0,1000	>0,1000	>0,1000
Passed normality test (alpha=0.05)?	Yes	Yes	Yes	Yes	Yes
P value summary	ns	ns	ns	ns	ns
Number of values	6	6	6	6	6

S2 Effect of the tested compounds on blood glucose levels in rats under glucose tolerance model (raw data).

S2.1 Control

Animal	Control_0h	Control_0.25h	Control_0.5h	Control_1h	Control_1.5h
1	4,7	10,3	10,9	10,3	7,1
2	4,8	10,6	11,2	10,1	8,2
3	4,1	10,7	10,8	9,6	6,9
4	5	11,2	11,6	10,4	7,5
5	4,5	10,5	10,2	9,7	7,4
6	4,4	11,2	11,1	10,7	7,5
7	4,6	10,8	10,9	10,5	6,8

Descriptive statistics of Control

	Control _0h	Control_0.25h	Control_0.5h	Control_1h	Control_1.5h
Number of values	7	7	7	7	7
Minimum	4,100	10,30	10,20	9,600	6,800
Maximum	5,000	11,20	11,60	10,70	8,200
Range	0,9000	0,9000	1,400	1,100	1,400
Mean	4,586	10,76	10,96	10,19	7,343
Std. Deviation	0,2911	0,3409	0,4276	0,4100	0,4721
Std. Error of Mean	0,1100	0,1288	0,1616	0,1550	0,1784

Test for normal distribution of Control

	Control_0h	Control_0.25h	Control_0.5h	Control_1h	Control_1.5h
Test for normal distribution					
Shapiro-Wilk test					
W	0,9918	0,9206	0,9516	0,9332	0,9225
P value	0,9961	0,4744	0,7442	0,5787	0,4887
Passed normality test (alpha=0.05)?	Yes	Yes	Yes	Yes	Yes
P value summary	ns	ns	ns	ns	ns
Kolmogorov-Smirnov test					
KS distance	0,1189	0,1888	0,2138	0,1812	0,2268
P value	>0,1000	>0,1000	>0,1000	>0,1000	>0,1000
Passed normality test (alpha=0.05)?	Yes	Yes	Yes	Yes	Yes
P value summary	ns	ns	ns	ns	ns
Number of values	7	7	7	7	7

S2.2 Metformin

Animal	Metformine_0h	Metformine_0.25h	Metformine_0.5h	Metformine_1h	Metformine_1,5h
1	5,4	11,6	9,4	9,3	6,4
2	4,6	12,2	10,7	8,1	6,1
3	6	12,9	13,8	10	8,8
4	5,3	11,7	11,2	9,1	7,4
5	6,1	13	14,1	10,3	8,9
6	4,5	11,3	8,5	7,9	5,4

Descriptive statistics of Metformin

	Metformine_0h	Metformine_0.25h	Metformine_0.5h	Metformine_1h	Metformine_1,5h
Number of values	6	6	6	6	6
Minimum	4,500	11,30	8,500	7,900	5,400
Maximum	6,100	13,00	14,10	10,30	8,900
Range	1,600	1,700	5,600	2,400	3,500
Mean	5,317	12,12	11,28	9,117	7,167
Std. Deviation	0,6735	0,7083	2,276	0,9725	1,454
Std. Error of Mean	0,2750	0,2892	0,9293	0,3970	0,5937

Test for normal distribution of Metformin

	Metformine_0h	Metformine_0.25h	Metformine_0.5h	Metformine_1h	Metformine_1,5h
Test for normal distribution					
Shapiro-Wilk test					
W	0,9022	0,8995	0,9191	0,9275	0,9069
P value	0,3873	0,3711	0,4992	0,5607	0,4162
Passed normality test (alpha=0.05)?	Yes	Yes	Yes	Yes	Yes
P value summary	ns	ns	ns	ns	ns
Kolmogorov-Smirnov test					
KS distance	0,1897	0,2218	0,1989	0,1854	0,2027
P value	>0,1000	>0,1000	>0,1000	>0,1000	>0,1000
Passed normality test (alpha=0.05)?	Yes	Yes	Yes	Yes	Yes
P value summary	ns	ns	ns	ns	ns
Number of values	6	6	6	6	6

S2.3 Gliclazide

Animal	Gliclazide_0h	Gliclazide_0.25h	Gliclazide_0.5h	Gliclazide_1h	Gliclazide_1,5h
1	3,3	6,3	8,8	9,2	6,5
2	4,7	9,8	13,3	14,9	6,6
3	3,8	6,7	7,6	8,4	5,8
4	4,3	7,1	9,6	9,2	5,4
5	4,9	10	13,5	14,8	7
6	3,1	5	6,1	6	5,2

Descriptive statistics of Gliclazide

	Gliclazide_0h	Gliclazide_0.25h	Gliclazide_0.5h	Gliclazide_1h	Gliclazide_1,5h
Number of values	6	6	6	6	6
Minimum	3,100	5,000	6,100	6,000	5,200
Maximum	4,900	10,00	13,50	14,90	7,000
Range	1,800	5,000	7,400	8,900	1,800
Mean	4,017	7,483	9,817	10,42	6,083
Std. Deviation	0,7387	2,001	3,017	3,629	0,7223
Std. Error of Mean	0,3016	0,8171	1,232	1,482	0,2949

Test for normal distribution of Gliclazide

	Gliclazide_0h	Gliclazide_0.25h	Gliclazide_0.5h	Gliclazide_1h	Gliclazide_1,5h
Test for normal distribution					
Shapiro-Wilk test					
W	0,9318	0,8945	0,9081	0,8586	0,9282
P value	0,5943	0,3424	0,4242	0,1843	0,5663
Passed normality test (alpha=0.05)?	Yes	Yes	Yes	Yes	Yes
P value summary	ns	ns	ns	ns	ns
Kolmogorov-Smirnov test					
KS distance	0,1674	0,2426	0,2092	0,2980	0,2180
P value	>0,1000	>0,1000	>0,1000	>0,1000	>0,1000
Passed normality test (alpha=0.05)?	Yes	Yes	Yes	Yes	Yes
P value summary	ns	ns	ns	ns	ns
Number of values	6	6	6	6	6

S2.4 Compound 3

Animal	Compound_3_0h	Compound_3_0.25h	Compound_3_0.5h	Compound_3_1h	Compound_3_1.5h
1	6,6	11,1	9,3	8,1	7,9
2	5,4	10,4	13	11,4	5,7
3	4,8	9,3	9,6	7,9	5,2
4	5,2	9,7	7,2	6,6	5,6
5	5,3	9,3	7,3	6,3	5
6	5,6	10,1	8,2	6,8	5,9

Descriptive statistics of Compound 3

	Compound_3_0h	Compound_3_0.25h	Compound_3_0.5h	Compound_3_1h	Compound_3_1.5h
Number of values	6	6	6	6	6
Minimum	4,800	9,300	7,200	6,300	5,000
Maximum	6,600	11,10	13,00	11,40	7,900
Range	1,800	1,800	5,800	5,100	2,900
Mean	5,483	9,983	9,100	7,850	5,883
Std. Deviation	0,6080	0,6998	2,152	1,883	1,042
Std. Error of Mean	0,2482	0,2857	0,8786	0,7689	0,4254

Test for normal distribution of Compound 3

	Compound_3_0h	Compound_3_0.25h	Compound_3_0.5h	Compound_3_1h	Compound_3_1.5h
Test for normal distribution					
Shapiro-Wilk test					
W	0,8843	0,9207	0,8635	0,8131	0,7893
P value	0,2893	0,5108	0,2014	0,0768	0,0470
Passed normality test (alpha=0.05)?	Yes	Yes	Yes	Yes	No
P value summary	ns	ns	ns	ns	*
Kolmogorov-Smirnov test					
KS distance	0,2572	0,1689	0,2415	0,2805	0,3270
P value	>0,1000	>0,1000	>0,1000	>0,1000	0,0440
Passed normality test (alpha=0.05)?	Yes	Yes	Yes	Yes	No
P value summary	ns	ns	ns	ns	*
Number of values	6	6	6	6	6

S2.6 Compound 17

Animal	Compound_17_0h	Compound_17_0.25h	Compound_17_0.5h	Compound_17_1h	Compound_17_1.5h
1	5,6	10,2	13,2	12,6	6,1
2	5,3	7,7	9,3	7,4	5,7
3	5,1	8,4	10,7	11,6	4,9
4	4,6	7,9	8,2	6,1	4,9
5	4,8	8,1	9,5	7,1	5
6	5,2	8,7	10,1	7,2	5,5

Descriptive statistics of Compound 17

	Compound_17_0h	Compound_17_0.25h	Compound_17_0.5h	Compound_17_1h	Compound_17_1.5h
Number of values	6	6	6	6	6
Minimum	4,600	7,700	8,200	6,100	4,900
Maximum	5,600	10,20	13,20	12,60	6,100
Range	1,000	2,500	5,000	6,500	1,200
Mean	5,100	8,500	10,17	8,667	5,350
Std. Deviation	0,3578	0,9055	1,706	2,716	0,4970
Std. Error of Mean	0,1461	0,3697	0,6965	1,109	0,2029

Test for normal distribution of Compound 17

	Compound_17_0h	Compound_17_0.25h	Compound_17_0.5h	Compound_17_1h	Compound_17_1.5h
Test for normal distribution					
Shapiro-Wilk test					
W	0,9805	0,8424	0,9209	0,8085	0,8762
P value	0,9539	0,1365	0,5115	0,0700	0,2522
Passed normality test (alpha=0.05)?	Yes	Yes	Yes	Yes	Yes
P value summary	ns	ns	ns	ns	ns
Kolmogorov-Smirnov test					
KS distance	0,1667	0,2459	0,2106	0,3462	0,2594
P value	>0,1000	>0,1000	>0,1000	0,0235	>0,1000
Passed normality test (alpha=0.05)?	Yes	Yes	Yes	No	Yes
P value summary	ns	ns	ns	*	ns
Number of values	6	6	6	6	6

S2.7 Compound 18

Animal	Compound_18_0h	Compound_18_0.25h	Compound_18_0.5h	Compound_18_h	Compound_18_1.5h
1	6,1	7,4	7,8	9,1	5,6
2	4,2	6,4	6,7	6,4	4,6
3	4,9	7,6	7,9	6,9	4,7
4	5,2	6,8	7,4	7,1	5
5	4,8	7,3	8,1	7,5	4,9
6	5,3	7,7	8,2	7,6	5

Descriptive statistics of Compound 18

	Compound_18_0h	Compound_18_0.25h	Compound_18_0.5h	Compound_18_h	Compound_18_1.5h
Number of values	6	6	6	6	6
Minimum	4,200	6,400	6,700	6,400	4,600
Maximum	6,100	7,700	8,200	9,100	5,600
Range	1,900	1,300	1,500	2,700	1,000
Mean	5,083	7,200	7,683	7,433	4,967
Std. Deviation	0,6306	0,5020	0,5565	0,9245	0,3502
Std. Error of Mean	0,2574	0,2049	0,2272	0,3774	0,1430

Test for normal distribution of Compound 18

	Compound_18_0h	Compound_18_0.25h	Compound_18_0.5h	Compound_18_h	Compound_18_1.5h
Test for normal distribution					
Shapiro-Wilk test					
W	0,9696	0,9074	0,8838	0,9062	0,8832
P value	0,8897	0,4195	0,2869	0,4116	0,2842
Passed normality test (alpha=0.05)?	Yes	Yes	Yes	Yes	Yes
P value summary	ns	ns	ns	ns	ns
Kolmogorov-Smirnov test					
KS distance	0,1989	0,2456	0,2497	0,2618	0,2954
P value	>0,1000	>0,1000	>0,1000	>0,1000	>0,1000
Passed normality test (alpha=0.05)?	Yes	Yes	Yes	Yes	Yes
P value summary	ns	ns	ns	ns	ns
Number of values	6	6	6	6	6

S2.8 Compound 20

Animal	Compound_20_0h	Compound_20_0.25h	Compound_20_0.5h	Compound_20_1h	Compound_20_1.5h
1	4,6	8,2	9,8	6,4	6,2
2	4,4	8,5	10,4	8,6	7,1
3	5	8,8	13	12,2	9,4
4	5,5	9,2	10,2	9,3	6,6
5	5	9,4	11,6	7,6	6
6	5,1	9,5	12,1	8,2	6,5

Descriptive statistics of Compound 20

	Compound_20_0h	Compound_20_0.25h	Compound_20_0.5h	Compound_20_1h	Compound_20_1.5h
Number of values	6	6	6	6	6
Minimum	4,400	8,200	9,800	6,400	6,000
Maximum	5,500	9,500	13,00	12,20	9,400
Range	1,100	1,300	3,200	5,800	3,400
Mean	4,933	8,933	11,18	8,717	6,967
Std. Deviation	0,3882	0,5203	1,250	1,968	1,250
Std. Error of Mean	0,1585	0,2124	0,5102	0,8035	0,5103

Test for normal distribution of Compound 20

	Compound_20_0h	Compound_20_0.25h	Compound_20_0.5h	Compound_20_1h	Compound_20_1.5h
Test for normal distribution					
Shapiro-Wilk test					
W	0,9537	0,9332	0,9314	0,9290	0,7667
P value	0,7703	0,6051	0,5908	0,5721	0,0289
Passed normality test (alpha=0.05)?	Yes	Yes	Yes	Yes	No
P value summary	ns	ns	ns	ns	*
Kolmogorov-Smirnov test					
KS distance	0,2349	0,1959	0,2346	0,2168	0,2909
P value	>0,1000	>0,1000	>0,1000	>0,1000	>0,1000
Passed normality test (alpha=0.05)?	Yes	Yes	Yes	Yes	Yes
P value summary	ns	ns	ns	ns	ns
Number of values	6	6	6	6	6

S2.9 Mann-Whitney test of Glucose tolerance test

Metformine_0h vs Control_0h

Mann Whitney test	
P value	0,0682
Exact or approximate P value?	Exact
P value summary	ns
Significantly different (P < 0.05)?	No
One- or two-tailed P value?	Two-tailed
Sum of ranks in column A,K	36 , 55
Mann-Whitney U	8
Difference between medians	
Median of column A	4,600, n=7
Median of column K	5,350, n=6
Difference: Actual	0,7500
Difference: Hodges-Lehmann	0,7500

Gliclazide_0h vs Control_0h

Mann Whitney test	
P value	0,1929
Exact or approximate P value?	Exact
P value summary	ns
Significantly different (P < 0.05)?	No
One- or two-tailed P value?	Two-tailed
Sum of ranks in column A,P	58,50 , 32,50
Mann-Whitney U	11,50
Difference between medians	
Median of column A	4,600, n=7
Median of column P	4,050, n=6
Difference: Actual	-0,5500
Difference: Hodges-Lehmann	-0,5500

Compound_3_0h vs Control_0h

Mann Whitney test	
P value	0,0029
Exact or approximate P value?	Exact
P value summary	**

Significantly different (P < 0.05)?	Yes
One- or two-tailed P value?	Two-tailed
Sum of ranks in column A,U	29,50 , 61,50
Mann-Whitney U	1,500
Difference between medians	
Median of column A	4,600, n=7
Median of column U	5,350, n=6
Difference: Actual	0,7500
Difference: Hodges-Lehmann	0,8000

Compound_17_0h vs Control_0h

Mann Whitney test	
P value	0,0198
Exact or approximate P value?	Exact
P value summary	*
Significantly different (P < 0.05)?	Yes
One- or two-tailed P value?	Two-tailed
Sum of ranks in column A,Z	33 , 58
Mann-Whitney U	5

Difference between medians	
Median of column A	4,600, n=7
Median of column Z	5,150, n=6
Difference: Actual	0,5500
Difference: Hodges-Lehmann	0,5000

Compound_18_0h vs Control_0h

Mann Whitney test	
P value	0,0804
Exact or approximate P value?	Exact
P value summary	ns
Significantly different (P < 0.05)?	No
One- or two-tailed P value?	Two-tailed
Sum of ranks in column A,AE	36,50 , 54,50
Mann-Whitney U	8,500
Difference between medians	
Median of column A	4,600, n=7
Median of column AE	5,050, n=6
Difference: Actual	0,4500

Difference: Hodges-Lehmann	0,4500

Compound_20_0h vs Control_0h

Mann Whitney test	
P value	0,1241
Exact or approximate P value?	Exact
P value summary	ns
Significantly different (P < 0.05)?	No
One- or two-tailed P value?	Two-tailed
Sum of ranks in column A,AJ	38 , 53
Mann-Whitney U	10
Difference between medians	
Median of column A	4,600, n=7
Median of column AJ	5,000, n=6
Difference: Actual	0,4000
Difference: Hodges-Lehmann	0,3500

Metformine_0,25h vs Control_0,25h

Mann Whitney test	
P value	0,0006
Exact or approximate P value?	Exact
P value summary	***
Significantly different (P < 0.05)?	Yes
One- or two-tailed P value?	Two-tailed
Sum of ranks in column B,L	28 , 63
Mann-Whitney U	0
Difference between medians	
Median of column B	10,70, n=7
Median of column L	11,95, n=6
Difference: Actual	1,250
Difference: Hodges-Lehmann	1,250

Gliclazide_0,25h vs Control_0,25h

Mann Whitney test	
P value	0,0012
Exact or approximate P value?	Exact
P value summary	**

Significantly different (P < 0.05)?	Yes
One- or two-tailed P value?	Two-tailed
Sum of ranks in column B,Q	70 , 21
Mann-Whitney U	0
Difference between medians	
Median of column B	10,70, n=7
Median of column Q	6,900, n=6
Difference: Actual	-3,800
Difference: Hodges-Lehmann	-3,850

Compound_3_0,25h vs Control_0,25h

Mann Whitney test	
P value	0,0326
Exact or approximate P value?	Exact
P value summary	*
Significantly different (P < 0.05)?	Yes
One- or two-tailed P value?	Two-tailed
Sum of ranks in column B,V	64 , 27
Mann-Whitney U	6

Difference between medians	
Median of column B	10,70, n=7
Median of column V	9,900, n=6
Difference: Actual	-0,8000
Difference: Hodges-Lehmann	-0,8500

Compound_17_0,25h vs Control_0,25h

Mann Whitney test	
P value	0,0012
Exact or approximate P value?	Exact
P value summary	**
Significantly different (P < 0.05)?	Yes
One- or two-tailed P value?	Two-tailed
Sum of ranks in column B,AA	70 , 21
Mann-Whitney U	0
Difference between medians	
Median of column B	10,70, n=7
Median of column AA	8,250, n=6
Difference: Actual	-2,450

Difference: Hodges-Lehmann	-2,500

Compound_18_0,25h vs Control_0,25h

Mann Whitney test	
P value	0,0012
Exact or approximate P value?	Exact
P value summary	**
Significantly different (P < 0.05)?	Yes
One- or two-tailed P value?	Two-tailed
Sum of ranks in column B,AF	70 , 21
Mann-Whitney U	0
Difference between medians	
Median of column B	10,70, n=7
Median of column AF	7,350, n=6
Difference: Actual	-3,350
Difference: Hodges-Lehmann	-3,500

Compound_20_0,25h vs Control_0,25h

Mann Whitney test

P value	0,0012
Exact or approximate P value?	Exact
P value summary	**
Significantly different (P < 0.05)?	Yes
One- or two-tailed P value?	Two-tailed
Sum of ranks in column B,AK	70 , 21
Mann-Whitney U	0
Difference between medians	
Median of column B	10,70, n=7
Median of column AK	9,000, n=6
Difference: Actual	-1,700
Difference: Hodges-Lehmann	-1,800

Metformine_0,5h vs Control_0,5h

Mann Whitney test	
P value	0,9720
Exact or approximate P value?	Exact
P value summary	ns
Significantly different (P < 0.05)?	No
One- or two-tailed P value?	Two-tailed

Sum of ranks in column C,M	49,50 , 41,50
Mann-Whitney U	20,50
Difference between medians	
Median of column C	10,90, n=7
Median of column M	10,95, n=6
Difference: Actual	0,05000
Difference: Hodges-Lehmann	-0,05000

Gliclazide_0,5h vs Control_0,5h

Mann Whitney test	
P value	0,3456
Exact or approximate P value?	Exact
P value summary	ns
Significantly different (P < 0.05)?	No
One- or two-tailed P value?	Two-tailed
Sum of ranks in column C,R	56 , 35
Mann-Whitney U	14
Difference between medians	
Median of column C	10,90, n=7

Median of column R	9,200, n=6
Difference: Actual	-1,700
Difference: Hodges-Lehmann	-1,800

Compound_3_0,5h vs Control_0,5h

Mann Whitney test	
P value	0,0460
Exact or approximate P value?	Exact
P value summary	*
Significantly different (P < 0.05)?	Yes
One- or two-tailed P value?	Two-tailed
Sum of ranks in column C,W	63 , 28
Mann-Whitney U	7
Difference between medians	
Median of column C	10,90, n=7
Median of column W	8,750, n=6
Difference: Actual	-2,150
Difference: Hodges-Lehmann	-2,150

Compound_17_0,5h vs Control_0,5h

Mann Whitney test	
P value	0,0670
Exact or approximate P value?	Exact
P value summary	ns
Significantly different (P < 0.05)?	No
One- or two-tailed P value?	Two-tailed
Sum of ranks in column C,AB	62 , 29
Mann-Whitney U	8
Difference between medians	
Median of column C	10,90, n=7
Median of column AB	9,800, n=6
Difference: Actual	-1,100
Difference: Hodges-Lehmann	-1,050

Compound_18_0,5h vs Control_0,5h

Mann Whitney test	
P value	0,0012
Exact or approximate P value?	Exact
P value summary	**

Significantly different (P < 0.05)?	Yes
One- or two-tailed P value?	Two-tailed
Sum of ranks in column C,AG	70 , 21
Mann-Whitney U	0
Difference between medians	
Median of column C	10,90, n=7
Median of column AG	7,850, n=6
Difference: Actual	-3,050
Difference: Hodges-Lehmann	-3,150

Compound_20_0,5h vs Control_0,5h

Mann Whitney test	
P value	0,9155
Exact or approximate P value?	Exact
P value summary	ns
Significantly different (P < 0.05)?	No
One- or two-tailed P value?	Two-tailed
Sum of ranks in column C,AL	48 , 43
Mann-Whitney U	20

Difference between medians	
Median of column C	10,90, n=7
Median of column AL	11,00, n=6
Difference: Actual	0,1000
Difference: Hodges-Lehmann	0,1000

Metformine_1h vs Control_1h

Mann Whitney test	
P value	0,0245
Exact or approximate P value?	Exact
P value summary	*
Significantly different (P < 0.05)?	Yes
One- or two-tailed P value?	Two-tailed
Sum of ranks in column D,N	64,50 , 26,50
Mann-Whitney U	5,500
Difference between medians	
Median of column D	10,30, n=7
Median of column N	9,200, n=6
Difference: Actual	-1,100

Difference: Hodges-Lehmann	-1,050

Gliclazide_1h vs Control_1h

Mann Whitney test	
P value	0,3456
Exact or approximate P value?	Exact
P value summary	ns
Significantly different (P < 0.05)?	No
One- or two-tailed P value?	Two-tailed
Sum of ranks in column D,S	56 , 35
Mann-Whitney U	14
Difference between medians	
Median of column D	10,30, n=7
Median of column S	9,200, n=6
Difference: Actual	-1,100
Difference: Hodges-Lehmann	-1,100

Compound_3_1h vs Control_1h

Mann Whitney test	
P value	0,0513
Exact or approximate P value?	Exact
P value summary	ns
Significantly different (P < 0.05)?	No
One- or two-tailed P value?	Two-tailed
Sum of ranks in column D,X	63 , 28
Mann-Whitney U	7
Difference between medians	
Median of column D	10,30, n=7
Median of column X	7,350, n=6
Difference: Actual	-2,950
Difference: Hodges-Lehmann	-2,800

Compound_17_1h vs Control_1h

Mann Whitney test	
P value	0,3660
Exact or approximate P value?	Exact
P value summary	ns

Significantly different (P < 0.05)?	No
One- or two-tailed P value?	Two-tailed
Sum of ranks in column D,AC	56 , 35
Mann-Whitney U	14
Difference between medians	
Median of column D	10,30, n=7
Median of column AC	7,300, n=6
Difference: Actual	-3,000
Difference: Hodges-Lehmann	-2,800

Compound_18_h vs Control_1h

Mann Whitney test	
P value	0,0012
Exact or approximate P value?	Exact
P value summary	**
Significantly different (P < 0.05)?	Yes
One- or two-tailed P value?	Two-tailed
Sum of ranks in column D,AH	70 , 21

Mann-Whitney U	0
Difference between medians	
Median of column D	10,30, n=7
Median of column AH	7,300, n=6
Difference: Actual	-3,000
Difference: Hodges-Lehmann	-2,900

Compound_20_1h vs Control_1h

Mann Whitney test	
P value	0,0513
Exact or approximate P value?	Exact
P value summary	ns
Significantly different (P < 0.05)?	No
One- or two-tailed P value?	Two-tailed
Sum of ranks in column D,AM	63 , 28
Mann-Whitney U	7
Difference between medians	
Median of column D	10,30, n=7
Median of column AM	8,400, n=6

Difference: Actual	-1,900
Difference: Hodges-Lehmann	-1,850

Metformine_1,5h vs Control_1,5h

Mann Whitney test	
P value	0,6503
Exact or approximate P value?	Exact
P value summary	ns
Significantly different (P < 0.05)?	No
One- or two-tailed P value?	Two-tailed
Sum of ranks in column E,O	52,50 , 38,50
Mann-Whitney U	17,50
Difference between medians	
Median of column E	7,400, n=7
Median of column O	6,900, n=6
Difference: Actual	-0,5000
Difference: Hodges-Lehmann	-0,4500

Gliclazide_1,5h vs Control_1,5h

Mann Whitney test	
P value	0,0047
Exact or approximate P value?	Exact
P value summary	**
Significantly different (P < 0.05)?	Yes
One- or two-tailed P value?	Two-tailed
Sum of ranks in column E,T	68 , 23
Mann-Whitney U	2
Difference between medians	
Median of column E	7,400, n=7
Median of column T	6,150, n=6
Difference: Actual	-1,250
Difference: Hodges-Lehmann	-1,250

Compound_3_1,5h vs Control_1,5h

Mann Whitney test	
P value	0,0321
Exact or approximate P value?	Exact
P value summary	*

Significantly different (P < 0.05)?	Yes
One- or two-tailed P value?	Two-tailed
Sum of ranks in column E,Y	64 , 27
Mann-Whitney U	6
Difference between medians	
Median of column E	7,400, n=7
Median of column Y	5,650, n=6
Difference: Actual	-1,750
Difference: Hodges-Lehmann	-1,700

Compound_17_1,5h vs Control_1,5h

Mann Whitney test	
P value	0,0012
Exact or approximate P value?	Exact
P value summary	**
Significantly different (P < 0.05)?	Yes
One- or two-tailed P value?	Two-tailed
Sum of ranks in column E,AD	70 , 21
Mann-Whitney U	0

Difference between medians	
Median of column E	7,400, n=7
Median of column AD	5,250, n=6
Difference: Actual	-2,150
Difference: Hodges-Lehmann	-2,000

Compound_18_1,5h vs Control_1,5h

Mann Whitney test	
P value	0,0012
Exact or approximate P value?	Exact
P value summary	**
Significantly different (P < 0.05)?	Yes
One- or two-tailed P value?	Two-tailed
Sum of ranks in column E,AI	70 , 21
Mann-Whitney U	0
Difference between medians	
Median of column E	7,400, n=7
Median of column AI	4,950, n=6
Difference: Actual	-2,450

Difference: Hodges-Lehmann	-2,400

Compound_20_1,5h vs Control_1,5h

Mann Whitney test	
P value	0,1090
Exact or approximate P value?	Exact
P value summary	ns
Significantly different (P < 0.05)?	No
One- or two-tailed P value?	Two-tailed
Sum of ranks in column E,AN	60,50 , 30,50
Mann-Whitney U	9,500
Difference between medians	
Median of column E	7,400, n=7
Median of column AN	6,550, n=6
Difference: Actual	-0,8500
Difference: Hodges-Lehmann	-0,7500

S3 Effect of the tested compounds on blood glucose levels in rats under adrenaline test (raw data).

S3.1 Control

Animal	Control_0h	Control_0.5h	Control_1.5h
1	4,9	12,6	22,4
2	4,9	13,3	23
3	6,3	13,1	21,9
4	4,8	11,5	20,2
5	4,1	9,6	20,6
6	5,3	11,9	21,3

Descriptive statistics of Control

	Control_0h	Control_0.5h	Control_1.5h
Number of values	6	6	6
Minimum	4,100	9,600	20,20
Maximum	6,300	13,30	23,00
Range	2,200	3,700	2,800
Mean	5,050	12,00	21,57
Std. Deviation	0,7259	1,362	1,071
Std. Error of Mean	0,2964	0,5562	0,4372

Test for normal distribution of Control

	Control_0h	Control_0.5h	Control_1.5h
Test for normal distribution			
Shapiro-Wilk test			
W	0,9175	0,9000	0,9703
P value	0,4878	0,3737	0,8941
Passed normality test (alpha=0.05)?	Yes	Yes	Yes
P value summary	ns	ns	ns
Kolmogorov-Smirnov test			
KS distance	0,2485	0,1901	0,1500
P value	>0,1000	>0,1000	>0,1000
Passed normality test (alpha=0.05)?	Yes	Yes	Yes
P value summary	ns	ns	ns
Number of values	6	6	6

S3.2 Metformin

Animal	Metformin_0.5h	Metformin_1.5h
1	6,4	10,3
2	5,9	9,8
3	6,7	11,4
4	7,3	11,2
5	7,4	11,6
6	6,9	10,2

Descriptive statistics of Metformin

	Metformin_0h	Metformin_0.5h	Metformin_1.5h
Number of values	6	6	6
Minimum	4,800	5,900	9,800
Maximum	6,300	7,400	11,60
Range	1,500	1,500	1,800
Mean	5,650	6,767	10,75
Std. Deviation	0,5788	0,5645	0,7423
Std. Error of Mean	0,2363	0,2305	0,3030

Test for normal distribution of Metformin

	Metformin_0h	Metformin_0.5h	Metformin_1.5h
Test for normal distribution			
Shapiro-Wilk test			
W	0,9521	0,9569	0,8988
P value	0,7570	0,7953	0,3671
Passed normality test (alpha=0.05)?	Yes	Yes	Yes
P value summary	ns	ns	ns
Kolmogorov-Smirnov test			
KS distance	0,1623	0,1609	0,2278
P value	>0,1000	>0,1000	>0,1000
Passed normality test (alpha=0.05)?	Yes	Yes	Yes
P value summary	ns	ns	ns
Number of values	6	6	6

S3.3 Gliclazide

Animal	Gliclazide_0h	Gliclazide_0.5h	Gliclazide_1.5h
1	6,7	8,4	11,1
2	5,9	7,4	10,5
3	6,4	8,1	10,4
4	6,8	8,5	11,6
5	5,7	7,2	9,5
6	6,2	7,7	9,8

Descriptive statistics of Gliclazide

	Gliclazide_0h	Gliclazide_0.5h	Gliclazide_1.5h
Number of values	6	6	6
Minimum	5,700	7,200	9,500
Maximum	6,800	8,500	11,60
Range	1,100	1,300	2,100
Mean	6,283	7,883	10,48
Std. Deviation	0,4355	0,5345	0,7834
Std. Error of Mean	0,1778	0,2182	0,3198

Test for normal distribution of Gliclazide

	Gliclazide_0h	Gliclazide_0.5h	Gliclazide_1.5h
Test for normal distribution			
Shapiro-Wilk test			
W	0,9506	0,9289	0,9687
P value	0,7455	0,5718	0,8833
Passed normality test (alpha=0.05)?	Yes	Yes	Yes
P value summary	ns	ns	ns
Kolmogorov-Smirnov test			
KS distance	0,1640	0,1665	0,1582
P value	>0,1000	>0,1000	>0,1000
Passed normality test (alpha=0.05)?	Yes	Yes	Yes
P value summary	ns	ns	ns
Number of values	6	6	6

S3.4 Compound 17

Animal	Compound_17_0h	Compound_17_0.5h	Compound_17_1.5h
1	4,5	9,3	22,1
2	5,4	13,8	19,5
3	5,1	13,2	19,9
4	5,2	10,3	20,5
5	4,9	11,4	19,8
6	5	11,8	21,3

Descriptive statistics of Compound 17

	Compound_17_0h	Compound_17_0.5h	Compound_17_1.5h
Number of values	6	6	6
Minimum	4,500	9,300	19,50
Maximum	5,400	13,80	22,10
Range	0,9000	4,500	2,600
Mean	5,017	11,63	20,52
Std. Deviation	0,3061	1,700	1,005
Std. Error of Mean	0,1249	0,6941	0,4102

Test for normal distribution of Compound 17

Test for normal distribution			
Shapiro-Wilk test			
W	0,9634	0,9692	0,9120
P value	0,8456	0,8870	0,4499
Passed normality test (alpha=0.05)?	Yes	Yes	Yes
P value summary	ns	ns	ns
Kolmogorov-Smirnov test			
KS distance	0,1849	0,1549	0,2303
P value	>0,1000	>0,1000	>0,1000
Passed normality test (alpha=0.05)?	Yes	Yes	Yes
P value summary	ns	ns	ns
Number of values	6	6	6

S3.5 Compound 18

Animal	Compound_18_0h	Compound_18_0.5h	Compound_18_1.5h
1	4,9	14,8	22,2
2	4,7	12,4	16,7
3	4,9	13,1	17,8
4	5,1	14,1	18,1
5	4,9	12,8	20,2
6	5,2	13,2	20,5

Descriptive statistics of Compound 18

	Compound_18_0h	Compound_18_0.5h	Compound_18_1.5h
Number of values	6	6	6
Minimum	4,700	12,40	16,70
Maximum	5,200	14,80	22,20
Range	0,5000	2,400	5,500
Mean	4,950	13,40	19,25
Std. Deviation	0,1761	0,8877	2,054
Std. Error of Mean	0,07188	0,3624	0,8386

Test for normal distribution of Compound 18

	Compound_18_0h	Compound_18_0.5h	Compound_18_1.5h
Test for normal distribution			
Shapiro-Wilk test			
W	0,9200	0,9326	0,9513
P value	0,5055	0,6003	0,7505
Passed normality test (alpha=0.05)?	Yes	Yes	Yes
P value summary	ns	ns	ns
Kolmogorov-Smirnov test			
KS distance	0,2785	0,2558	0,2122
P value	>0,1000	>0,1000	>0,1000
Passed normality test (alpha=0.05)?	Yes	Yes	Yes
P value summary	ns	ns	ns
Number of values	6	6	6

S3.6 Compound 20

Animal	Compound_20_0h	Compound_20_0,5h	Compound_20_1,5h
1	4,9	9,9	16
2	6	12,9	14,8
3	4,9	13,8	17,7
4	5,6	12,2	19,3
5	4,6	11,2	17,3
6	4,8	12,2	17,1

Descriptive statistics of Compound 20

	Compound_20_0h	Compound_20_0,5h	Compound_20_1,5h
Number of values	6	6	6
Minimum	4,600	9,900	14,80
Maximum	6,000	13,80	19,30
Range	1,400	3,900	4,500
Mean	5,133	12,03	17,03
Std. Deviation	0,5428	1,354	1,531
Std. Error of Mean	0,2216	0,5530	0,6249

Test for normal distribution of Compound 20

	Compound_20_0h	Compound_20_0,5h	Compound_20_1,5h
Test for normal distribution			
Shapiro-Wilk test			
W	0,8586	0,9712	0,9801
P value	0,1844	0,9003	0,9520
Passed normality test (alpha=0.05)?	Yes	Yes	Yes
P value summary	ns	ns	ns
Kolmogorov-Smirnov test			
KS distance	0,3330	0,2156	0,1840
P value	0,0363	>0,1000	>0,1000
Passed normality test (alpha=0.05)?	No	Yes	Yes
P value summary	*	ns	ns
Number of values	6	6	6

S3.7 Mann-Whitney test of adrenaline test

Metformin_0h vs Control_0h

Mann Whitney test	
P value	0,2294
Exact or approximate P value?	Exact
P value summary	ns
Significantly different (P < 0.05)?	No
One- or two-tailed P value?	Two-tailed
Sum of ranks in column A,G	31 , 47
Mann-Whitney U	10
Difference between medians	
Median of column A	4,900, n=6
Median of column G	5,700, n=6
Difference: Actual	0,8000
Difference: Hodges-Lehmann	0,7000

Gliclazide_0h vs Control_0h

Mann Whitney test

P value	0,0130
Exact or approximate P value?	Exact
P value summary	*
Significantly different (P < 0.05)?	Yes
One- or two-tailed P value?	Two-tailed
Sum of ranks in column A,J	24 , 54
Mann-Whitney U	3
Difference between medians	
Median of column A	4,900, n=6
Median of column J	6,300, n=6
Difference: Actual	1,400
Difference: Hodges-Lehmann	1,350

Compound_17_0h vs Control_0h

Mann Whitney test	
P value	0,6970
Exact or approximate P value?	Exact
P value summary	ns
Significantly different (P < 0.05)?	No
One- or two-tailed P value?	Two-tailed

Sum of ranks in column A,M	36 , 42
Mann-Whitney U	15
Difference between medians	
Median of column A	4,900, n=6
Median of column M	5,050, n=6
Difference: Actual	0,1500
Difference: Hodges-Lehmann	0,1000

Compound_18_0h vs Control_0h

Mann Whitney test	
P value	>0,9999
Exact or approximate P value?	Exact
P value summary	ns
Significantly different (P < 0.05)?	No
One- or two-tailed P value?	Two-tailed
Sum of ranks in column A,P	39 , 39
Mann-Whitney U	18
Difference between medians	
Median of column A	4,900, n=6
Median of column P	4,900, n=6

Difference: Actual	0,000
Difference: Hodges-Lehmann	0,000

Compound_20_0h vs Control_0h

Mann Whitney test	
P value	0,5076
Exact or approximate P value?	Exact
P value summary	ns
Significantly different (P < 0.05)?	No
One- or two-tailed P value?	Two-tailed
Sum of ranks in column D,S	54 , 37
Mann-Whitney U	16
Difference between medians	
Median of column D	5,200, n=7
Median of column S	4,900, n=6
Difference: Actual	-0,3000
Difference: Hodges-Lehmann	-0,2000

Metformin_0,5h vs Control_0,5h

Mann Whitney test

P value	0,0022
Exact or approximate P value?	Exact
P value summary	**
Significantly different (P < 0.05)?	Yes
One- or two-tailed P value?	Two-tailed
Sum of ranks in column B,H	57 , 21
Mann-Whitney U	0
Difference between medians	
Median of column B	12,25, n=6
Median of column H	6,800, n=6
Difference: Actual	-5,450
Difference: Hodges-Lehmann	-5,550

Gliclazide_0,5h vs Control_0,5h

Mann Whitney test	
P value	0,0022
Exact or approximate P value?	Exact
P value summary	**
Significantly different (P < 0.05)?	Yes
One- or two-tailed P value?	Two-tailed

Sum of ranks in column B,K	57 , 21
Mann-Whitney U	0
Difference between medians	
Median of column B	12,25, n=6
Median of column K	7,900, n=6
Difference: Actual	-4,350
Difference: Hodges-Lehmann	-4,400

Compound_17_0,5h vs Control_0,5h

Mann Whitney test	
P value	0,6991
Exact or approximate P value?	Exact
P value summary	ns
Significantly different (P < 0.05)?	No
One- or two-tailed P value?	Two-tailed
Sum of ranks in column B,N	42 , 36
Mann-Whitney U	15
Difference between medians	
Median of column B	12,25, n=6
Median of column N	11,60, n=6

Difference: Actual	-0,6500
Difference: Hodges-Lehmann	-0,2000

Compound_18_0,5h vs Control_0,5h

Mann Whitney test	
P value	0,1017
Exact or approximate P value?	Exact
P value summary	ns
Significantly different (P < 0.05)?	No
One- or two-tailed P value?	Two-tailed
Sum of ranks in column B,Q	28,50 , 49,50
Mann-Whitney U	7,500
Difference between medians	
Median of column B	12,25, n=6
Median of column Q	13,15, n=6
Difference: Actual	0,9000
Difference: Hodges-Lehmann	1,250

Compound_20_0,5h vs Control_0,5h

Mann Whitney test

P value	>0,9999
Exact or approximate P value?	Exact
P value summary	ns
Significantly different (P < 0.05)?	No
One- or two-tailed P value?	Two-tailed
Sum of ranks in column B,T	39 , 39
Mann-Whitney U	18
Difference between medians	
Median of column B	12,25, n=6
Median of column T	12,20, n=6
Difference: Actual	-0,05000
Difference: Hodges-Lehmann	0,05000

Metformin_1,5h vs Control_1,5h

Mann Whitney test	
P value	0,0022
Exact or approximate P value?	Exact
P value summary	**
Significantly different (P < 0.05)?	Yes
One- or two-tailed P value?	Two-tailed
Sum of ranks in column C,I	57 , 21

Mann-Whitney U	0
Difference between medians	
Median of column C	21,60, n=6
Median of column I	10,75, n=6
Difference: Actual	-10,85
Difference: Hodges-Lehmann	-10,80

Gliclazide_1,5h vs Control_1,5h

Mann Whitney test	
P value	0,0022
Exact or approximate P value?	Exact
P value summary	**
Significantly different (P < 0.05)?	Yes
One- or two-tailed P value?	Two-tailed
Sum of ranks in column C,L	57 , 21
Mann-Whitney U	0
Difference between medians	
Median of column C	21,60, n=6
Median of column L	10,45, n=6
Difference: Actual	-11,15

Difference: Hodges-Lehmann	-11,00

Compound_17_1,5h vs Control_1,5h

Mann Whitney test	
P value	0,1017
Exact or approximate P value?	Exact
P value summary	ns
Significantly different (P < 0.05)?	No
One- or two-tailed P value?	Two-tailed
Sum of ranks in column C,O	49,50 , 28,50
Mann-Whitney U	7,500
Difference between medians	
Median of column C	21,60, n=6
Median of column O	20,20, n=6
Difference: Actual	-1,400
Difference: Hodges-Lehmann	-1,000

Compound_18_1,5h vs Control_1,5h

Mann Whitney test

P value	0,0455
Exact or approximate P value?	Exact
P value summary	*
Significantly different (P < 0.05)?	Yes
One- or two-tailed P value?	Two-tailed
Sum of ranks in column C,R	51,50 , 26,50
Mann-Whitney U	5,500
Difference between medians	
Median of column C	21,60, n=6
Median of column R	19,15, n=6
Difference: Actual	-2,450
Difference: Hodges-Lehmann	-2,450

Compound_20_1,5h vs Control_1,5h

Mann Whitney test	
P value	0,0022
Exact or approximate P value?	Exact
P value summary	**
Significantly different (P < 0.05)?	Yes
One- or two-tailed P value?	Two-tailed

Sum of ranks in column C,U	57 , 21
Mann-Whitney U	0
Difference between medians	
Median of column C	21,60, n=6
Median of column U	17,20, n=6
Difference: Actual	-4,400
Difference: Hodges-Lehmann	-4,600

S4 Effect of the tested compounds on blood glucose levels in rats under insulin test (raw data).

S4.1 Control

Animal	Control_0h	Control_0.5h
1	5,5	2,8
2	5	2,4
3	4,9	2,7
4	4,2	2,4
5	4,7	2,6
6	5,2	2,8

Descriptive statistics of Control

	Control_0h	Control_0.5h
Number of values	6	6
Minimum	4,200	2,400
Maximum	5,500	2,800
Range	1,300	0,4000
Mean	4,917	2,617
Std. Deviation	0,4446	0,1835
Std. Error of Mean	0,1815	0,07491

Test for normal distribution of Control

	Control_0h	Control_0.5h
Test for normal distribution		
Shapiro-Wilk test		
W	0,9818	0,8502
P value	0,9600	0,1579
Passed normality test (alpha=0.05)?	Yes	Yes
P value summary	ns	ns
Kolmogorov-Smirnov test		
KS distance	0,1517	0,2145
P value	>0,1000	>0,1000
Passed normality test (alpha=0.05)?	Yes	Yes
P value summary	ns	ns
Number of values	6	6

S4.2 Metformin

Animal	Metformin_0h	Metformin_0.5h
1	5,6	4
2	4,7	2,5
3	4,9	3,6
4	5,4	3,7
5	5,8	4,3
6	4,6	2,6

Descriptive statistics of Metformin

	Metformin_0h	Metformin_0.5h
Number of values	6	6
Minimum	4,600	2,500
Maximum	5,800	4,300
Range	1,200	1,800
Mean	5,167	3,450
Std. Deviation	0,5007	0,7396
Std. Error of Mean	0,2044	0,3019

Test for normal distribution of Metformin

	Metformin_0h	Metformin_0.5h
Test for normal distribution		
Shapiro-Wilk test		
W	0,9106	0,8889
P value	0,4406	0,3122
Passed normality test (alpha=0.05)?	Yes	Yes
P value summary	ns	ns
Kolmogorov-Smirnov test		
KS distance	0,2029	0,2470
P value	>0,1000	>0,1000
Passed normality test (alpha=0.05)?	Yes	Yes
P value summary	ns	ns
Number of values	6	6

S4.3 Gliclazide

Animal	Gliclazide_0h	Gliclazide_0.5h
1	5,3	3,8
2	5,8	4,2
3	4,9	3,3
4	6,1	4,1
5	6,3	4,4
6	4,6	3,4

Descriptive statistics of Gliclazide

	Gliclazide_0h	Gliclazide_0.5h
Number of values	6	6
Minimum	4,600	3,300
Maximum	6,300	4,400
Range	1,700	1,100
Mean	5,500	3,867
Std. Deviation	0,6782	0,4457
Std. Error of Mean	0,2769	0,1820

Test for normal distribution of Gliclazide

	Gliclazide_0h	Gliclazide_0.5h
Test for normal distribution		
Shapiro-Wilk test		
W	0,9447	0,9245
P value	0,6971	0,5386
Passed normality test (alpha=0.05)?	Yes	Yes
P value summary	ns	ns
Kolmogorov-Smirnov test		
KS distance	0,1709	0,1997
P value	>0,1000	>0,1000
Passed normality test (alpha=0.05)?	Yes	Yes
P value summary	ns	ns
Number of values	6	6

S4.4 Compound 3

Compound 3

Animal	Compound_3_0h	Compound_3_0,5h
1	5,3	2,1
2	5	2,7
3	4,7	2,2
4	5,2	2,2
5	4,4	2,6
6	5,1	2,1

Descriptive statistics of Compound 3

	Compound_3_0h	Compound_3_0,5h
Number of values	6	6
Minimum	4,400	2,100
Maximum	5,300	2,700
Range	0,9000	0,6000
Mean	4,950	2,317
Std. Deviation	0,3391	0,2639
Std. Error of Mean	0,1384	0,1078

Test for normal distribution of Compound 3

	Compound_3_0h	Compound_3_0,5h
Test for normal distribution		
Shapiro-Wilk test		
W	0,9213	0,7947
P value	0,5151	0,0526
Passed normality test (alpha=0.05)?	Yes	Yes
P value summary	ns	ns
Kolmogorov-Smirnov test		
KS distance	0,2253	0,3374
P value	>0,1000	0,0315
Passed normality test (alpha=0.05)?	Yes	No
P value summary	ns	*
Number of values	6	6

S4.5 Compound 17

Animal	Compound_17_0h	Compound_17_0.5h
1	5,3	2,7
2	4,6	2,1
3	5,1	3,2
4	4,6	2,2
5	5	2,6
6	5,1	2,5

Descriptive statistics of Compound 17

	Compound_17_0h	Compound_17_0.5h
Number of values	6	6
Minimum	4,600	2,100
Maximum	5,300	3,200
Range	0,7000	1,100
Mean	4,950	2,550
Std. Deviation	0,2881	0,3937
Std. Error of Mean	0,1176	0,1607

Test for normal distribution of Compound 17

	Compound_17_0h	Compound_17_0.5h
Test for normal distribution		
Shapiro-Wilk test		
W	0,8653	0,9465
P value	0,2082	0,7115
Passed normality test (alpha=0.05)?	Yes	Yes
P value summary	ns	ns
Kolmogorov-Smirnov test		
KS distance	0,2356	0,1849
P value	>0,1000	>0,1000
Passed normality test (alpha=0.05)?	Yes	Yes
P value summary	ns	ns
Number of values	6	6

S4.6 Compound 18

Animal	Compound_18_0h	Compound_18_0.5h
1	5,7	2,7
2	3,7	2,6
3	5,2	3,4
4	4,9	2,9
5	5	2,4
6	5,3	2,6

Descriptive statistics of Compound 18

	Compound_18_0h	Compound_18_0.5h
Number of values	6	6
Minimum	3,700	2,400
Maximum	5,700	3,400
Range	2,000	1,000
Mean	4,967	2,767
Std. Deviation	0,6802	0,3502
Std. Error of Mean	0,2777	0,1430

Test for normal distribution of Compound 18

	Compound_18_0h	Compound_18_0.5h
Test for normal distribution		
Shapiro-Wilk test		
W	0,8666	0,8832
P value	0,2129	0,2842
Passed normality test (alpha=0.05)?	Yes	Yes
P value summary	ns	ns
Kolmogorov-Smirnov test		
KS distance	0,2943	0,2421
P value	>0,1000	>0,1000
Passed normality test (alpha=0.05)?	Yes	Yes
P value summary	ns	ns
Number of values	6	6

S4.7 Control

Animal	Compound_20_0h	Compound_20_0,5h
1	4,4	3,1
2	5	2,9
3	4,5	3,2
4	4,8	2,7
5	5,2	2,7
6	4,7	3

Descriptive statistics of I Control

	Compound_20_0h	Compound_20_0,5h
Number of values	6	6
Minimum	4,400	2,700
Maximum	5,200	3,200
Range	0,8000	0,5000
Mean	4,767	2,933
Std. Deviation	0,3011	0,2066
Std. Error of Mean	0,1229	0,08433

Test for normal distribution of Control

	Compound_20_0h	Compound_20_0,5h
Test for normal distribution		
Shapiro-Wilk test		
W	0,9713	0,9182
P value	0,9009	0,4928
Passed normality test (alpha=0.05)?	Yes	Yes
P value summary	ns	ns
Kolmogorov-Smirnov test		
KS distance	0,1454	0,2040
P value	>0,1000	>0,1000
Passed normality test (alpha=0.05)?	Yes	Yes
P value summary	ns	ns
Number of values	6	6

S4.8 Mann-Whitney test of insuline test

Metformin_0h vs Control_0h

Mann Whitney test	
P value	0,5584
Exact or approximate P value?	Exact
P value summary	ns
Significantly different (P < 0.05)?	No
One- or two-tailed P value?	Two-tailed
Sum of ranks in column A,E	35 , 43
Mann-Whitney U	14
Difference between medians	
Median of column A	4,950, n=6
Median of column E	5,150, n=6
Difference: Actual	0,2000
Difference: Hodges-Lehmann	0,2500

Gliclazide_0h vs Control_0h

Mann Whitney test

P value	0,1926
Exact or approximate P value?	Exact
P value summary	ns
Significantly different (P < 0.05)?	No
One- or two-tailed P value?	Two-tailed
Sum of ranks in column A,G	30,50 , 47,50
Mann-Whitney U	9,500
Difference between medians	
Median of column A	4,950, n=6
Median of column G	5,550, n=6
Difference: Actual	0,6000
Difference: Hodges-Lehmann	0,6000

Compound_3_0h vs Control_0h

Mann Whitney test	
P value	0,8658
Exact or approximate P value?	Exact
P value summary	ns
Significantly different (P < 0.05)?	No

One- or two-tailed P value?	Two-tailed
Sum of ranks in column A,I	37,50 , 40,50
Mann-Whitney U	16,50
Difference between medians	
Median of column A	4,950, n=6
Median of column I	5,050, n=6
Difference: Actual	0,1000
Difference: Hodges-Lehmann	0,05000

Compound_17_0h vs Control_0h

Mann Whitney test	
P value	0,9675
Exact or approximate P value?	Exact
P value summary	ns
Significantly different (P < 0.05)?	No
One- or two-tailed P value?	Two-tailed
Sum of ranks in column A,K	38,50 , 39,50
Mann-Whitney U	17,50
Difference between medians	

Median of column A	4,950, n=6
Median of column K	5,050, n=6
Difference: Actual	0,1000
Difference: Hodges-Lehmann	0,05000

Compound_18_0h vs Control_0h

Mann Whitney test	
P value	0,6234
Exact or approximate P value?	Exact
P value summary	ns
Significantly different (P < 0.05)?	No
One- or two-tailed P value?	Two-tailed
Sum of ranks in column A,K	35,50 , 42,50
Mann-Whitney U	14,50
Difference between medians	
Median of column A	4,950, n=6
Median of column K	5,100, n=6
Difference: Actual	0,1500
Difference: Hodges-Lehmann	0,1500

Compound_20_0h vs Control_0h

Mann Whitney test	
P value	0,5087
Exact or approximate P value?	Exact
P value summary	ns
Significantly different (P < 0.05)?	No
One- or two-tailed P value?	Two-tailed
Sum of ranks in column A,M	43,50 , 34,50
Mann-Whitney U	13,50
Difference between medians	
Median of column A	4,950, n=6
Median of column M	4,750, n=6
Difference: Actual	-0,2000
Difference: Hodges-Lehmann	-0,2000

Metformin_0,5h vs Control_0,5h

Mann Whitney test	
P value	0,1061
Exact or approximate P value?	Exact

P value summary	ns
Significantly different (P < 0.05)?	No
One- or two-tailed P value?	Two-tailed
Sum of ranks in column B,F	28,50 , 49,50
Mann-Whitney U	7,500
Difference between medians	
Median of column B	2,650, n=6
Median of column F	3,650, n=6
Difference: Actual	1,000
Difference: Hodges-Lehmann	1,000

Gliclazide_0,5h vs Control_0,5h

Mann Whitney test	
P value	0,0022
Exact or approximate P value?	Exact
P value summary	**
Significantly different (P < 0.05)?	Yes
One- or two-tailed P value?	Two-tailed
Sum of ranks in column B,H	21 , 57
Mann-Whitney U	0

Difference between medians	
Median of column B	2,650, n=6
Median of column H	3,950, n=6
Difference: Actual	1,300
Difference: Hodges-Lehmann	1,350

Compound_3_0,5h vs Control_0,5h

Mann Whitney test	
P value	0,0736
Exact or approximate P value?	Exact
P value summary	ns
Significantly different (P < 0.05)?	No
One- or two-tailed P value?	Two-tailed
Sum of ranks in column B,J	51 , 27
Mann-Whitney U	6
Difference between medians	
Median of column B	2,650, n=6
Median of column J	2,200, n=6
Difference: Actual	-0,4500

Difference: Hodges-Lehmann	-0,3000

Compound_17_0,5h vs Control_0,5h

Mann Whitney test	
P value	0,5584
Exact or approximate P value?	Exact
P value summary	ns
Significantly different (P < 0.05)?	No
One- or two-tailed P value?	Two-tailed
Sum of ranks in column B,J	43 , 35
Mann-Whitney U	14
Difference between medians	
Median of column B	2,650, n=6
Median of column J	2,550, n=6
Difference: Actual	-0,1000
Difference: Hodges-Lehmann	-0,1000

Compound_18_0,5h vs Control_0,5h

Mann Whitney test

P value	0,6234
Exact or approximate P value?	Exact
P value summary	ns
Significantly different (P < 0.05)?	No
One- or two-tailed P value?	Two-tailed
Sum of ranks in column B,L	35,50 , 42,50
Mann-Whitney U	14,50
Difference between medians	
Median of column B	2,650, n=6
Median of column L	2,650, n=6
Difference: Actual	0,000
Difference: Hodges-Lehmann	0,1000

Compound_20_0,5h vs Control_0,5h

Mann Whitney test	
P value	0,0390
Exact or approximate P value?	Exact
P value summary	*
Significantly different (P < 0.05)?	Yes
One- or two-tailed P value?	Two-tailed

Sum of ranks in column B,N	26 , 52
Mann-Whitney U	5
Difference between medians	
Median of column B	2,650, n=6
Median of column N	2,950, n=6
Difference: Actual	0,3000
Difference: Hodges-Lehmann	0,3000
